# Hydrophobic Gold Nanoparticles with Intrinsic Chirality for the Efficient Fabrication of Chiral Plasmonic Nanocomposites

**DOI:** 10.1021/acsami.2c11925

**Published:** 2022-10-28

**Authors:** Natalia Kowalska, Filip Bandalewicz, Jakub Kowalski, Sergio Gómez-Graña, Maciej Bagiński, Isabel Pastoriza-Santos, Marek Grzelczak, Joanna Matraszek, Jorge Pérez-Juste, Wiktor Lewandowski

**Affiliations:** † Laboratory of Organic Nanomaterials and Biomolecules, Faculty of Chemistry University of Warsaw, Pasteura 1 Street, 02-093 Warsaw, Poland; ‡ Departamento de Química Física, CINBIO, Universidade de Vigo, Campus Universitario As Lagoas, Marcosende, 36310 Vigo, Spain; § Instituto de Investigación Sanitaria Galicia Sur (IIS Galicia Sur), SERGAS-UVIGO, 36213 Vigo, Spain; ∥ Centro de Física de Materiales (CSIC-UPV/EHU) and Donostia International Physics Center, 20018 Donostia − San Sebastián, Spain

**Keywords:** supramolecular chirality, block copolymers, liquid crystals, gels, chiral metamolecules, reconfigurable nanostructures, circular dichroism, chirality transfer, phase transfer

## Abstract

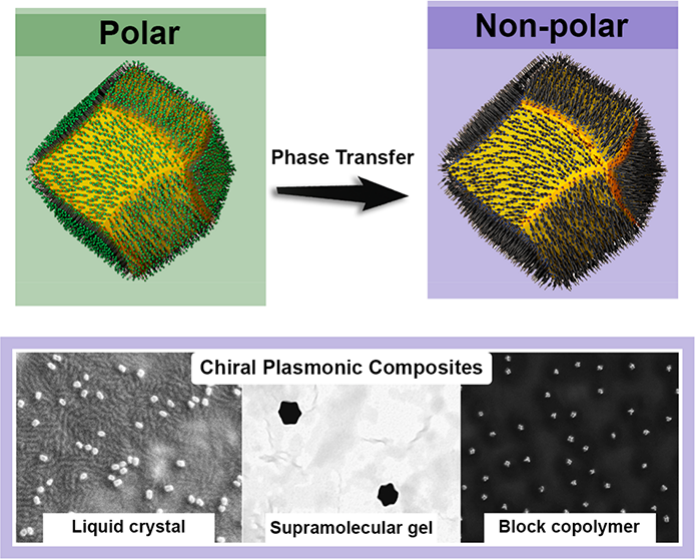

The development of plasmonic nanomaterials with chiral geometry has drawn extensive attention owing to their practical implications in chiral catalysis, chiral metamaterials, or enantioselective biosensing and medicine. However, due to the lack of effective synthesis methods of hydrophobic nanoparticles (NPs) showing intrinsic, plasmonic chirality, their applications are currently limited to aqueous systems. In this work, we resolve the problem of achieving hydrophobic Au NPs with intrinsic chirality by efficient phase transfer of water-soluble NPs using low molecular weight, liquid crystal-like ligands. We confirmed that, after the phase transfer, Au NPs preserve strong, far-field circular dichroism (CD) signals, attesting their chiral geometry. The universality of the method is exemplified by using different types of NPs and ligands. We further highlight the potential of the proposed approach to realize chiral plasmonic, inorganic/organic nanocomposites with block copolymers, liquid crystals, and compounds forming physical gels. All soft matter composites sustain plasmonic CD signals with electron microscopies confirming well-dispersed nanoinclusions. The developed methodology allows us to expand the portfolio of plasmonic NPs with intrinsic structural chirality, thereby broadening the scope of their applications toward soft-matter based systems.

## Introduction

Fascinating developments in the synthesis of plasmonic nanoparticles (NPs), the building blocks for emerging technologies of the 21st century,^1−4^ have recently taken a sudden twist.^5−8^ Inspired by nature, the use of amino acids and peptides enabled directing NPs growth at the atomic scale, leading to the bottom-up fabrication of NPs exhibiting chiral morphologies.^9−18^ The controlled handedness of intrinsically chiral plasmonic NPs and their strong light–matter interactions, allowed to advance the fields of biosensing,^19−23^ asymmetric photochemistry,^24^ asymmetric photophysics,^25^ biomimetic catalysis,^14^ and medicine.^26−29^ These unique innovations were unlocked by plasmonic circular dichroism (PCD) and chiral morphology of NPs. However, current applications of chiral plasmonic NPs are restricted to water-based systems since biomolecules are required for their synthesis.^30−34^ For example, a nanocomposite based on water-dispersible chiral gold NPs was used to prepare an ultrasensitive near-infrared circularly polarized light detection device.^35^ We propose that the development of hydrophobic, plasmonic NPs with intrinsic chirality should enable efficient and large-scale production of chiral organic–inorganic nanocomposites showing PCD properties, thereby providing crucial engineering advantages for future generations of soft matter-based functional devices.^36−40^

Among the different host materials explored in the context of chiral plasmonic composite fabrication, polymers, liquid crystals (LCs) and organic gels are particularly interesting.^41,42^ These composites can benefit from the synergy of plasmonic properties of NPs as well as the morphology and soft character of the template. For example, materials exhibiting stimuli-responsive PCD properties have been prepared through a directed assembly of NPs by chiral liquid crystals.^43,44^ However, remote control over the structure of LCs still remains a challenge. For example, the addition of intrinsically chiral nanoparticles, with chirally selective photothermal effect, could be used to fabricate devices exhibiting handedness of light-dependent phototunable dielectric permittivity.^45^ Composites exhibiting PCD properties, interesting for biosensing applications, were prepared using an anisotropic polymer matrix and proper stacking of composite layers,^37^ while actively tunable PCD structures were prepared by embedding NPs within gels.^46^ However, a strong limitation of this approach is that achieving PCD properties requires the use of host materials exhibiting chiral or anisotropic morphology, as all these composites were prepared using achiral NPs.^37^ Thus, to further increase the applicability of composite materials exhibiting PCD and ease their integration into flexible photonic devices, we propose to fabricate hydrophobic, plasmonic NPs with chiral morphology that can be interfaced with a broad spectrum of achiral hosts. It should be noted that recently, hydrophobic plasmonic NPs covered with chiral ligands were shown to induce the formation of chiral LC composites;^47,48^ however, these materials were not reported to exhibit PCD properties.

The last two decades have been the time of intense research on anisotropic plasmonic NPs, allowing us to amass in-depth knowledge of symmetry breaking processes and resulting in an exquisite control over the morphology of products. Noteworthy, almost all synthetic methods, including those toward chiral NPs, require aqueous media.^49,50^ Although the synthesis of morphologically chiral Au NPs in hydrophobic media seems plausible, given the well-developed asymmetric organic synthesis, currently, the only way to resolve the issue of availability of hydrophobic chiral NPs would be to synthesize chiral NPs in aqueous media and then perform a phase transfer. Along with this line, protocols and ligand designs were proposed to transfer hydrophilic, plasmonic NPs into hydrophobic media.^51−54^ However, producing colloidal dispersions of NPs larger than 100 nm, the usual size of morphologically chiral Au NPs, has proven challenging. If considering ligands of nonpolar nanoparticles, usually nonbranched, low molecular weight compounds, or high-molecular weight polymers were used.^49,55^ In the case of small particles, these ligands ensure relatively good colloidal stability; however, in the case of larger particles or phase transfer, they are not always efficient. Thus, to resolve these issues, the spectrum of ligands used was recently broadened to include new designs of organic shell of nanoparticles, e.g., branched alkyl thiols (called “entropic ligands”),^56^ binary organic shell of particles,^57^ or dendritic ligands comprising alkyl and aromatic parts.^58−62^ Thus, achieving nonpolar, colloidally stable intrinsically chiral, plasmonic NPs covered with a relatively thin monolayer of ligands and continuous development of ligands is a challenging yet rewarding goal.

In this contribution, we aimed at achieving colloidally stable chiral plasmonic NPs in hydrophobic media for the fabrication of nanocomposites exhibiting PCD properties. For this purpose, we synthesized Au NPs with chiral morphologies, following previously reported, slightly modified protocols (see the Materials and Methods section).^11,63^ The use of low-molecular weight thiols, comprising aromatic rings and alkyl chains, enabled the efficient transfer of Au NPs to an organic phase. Chiral properties and stability of the hydrophobic NPs were evidenced by far-field circular dichroism (CD) spectroscopy and scanning electron microscopy (SEM). To confirm the universality of the presented approach, dispersions of two types of chiral NPs were interfaced with block copolymers: liquid crystals and physical gels leading to nanocomposites exhibiting plasmonic chirality.

## Results and Discussion

### Synthesis and Phase Transfer of Intrinsically Chiral Gold Nanoparticles

**Figure 1:**
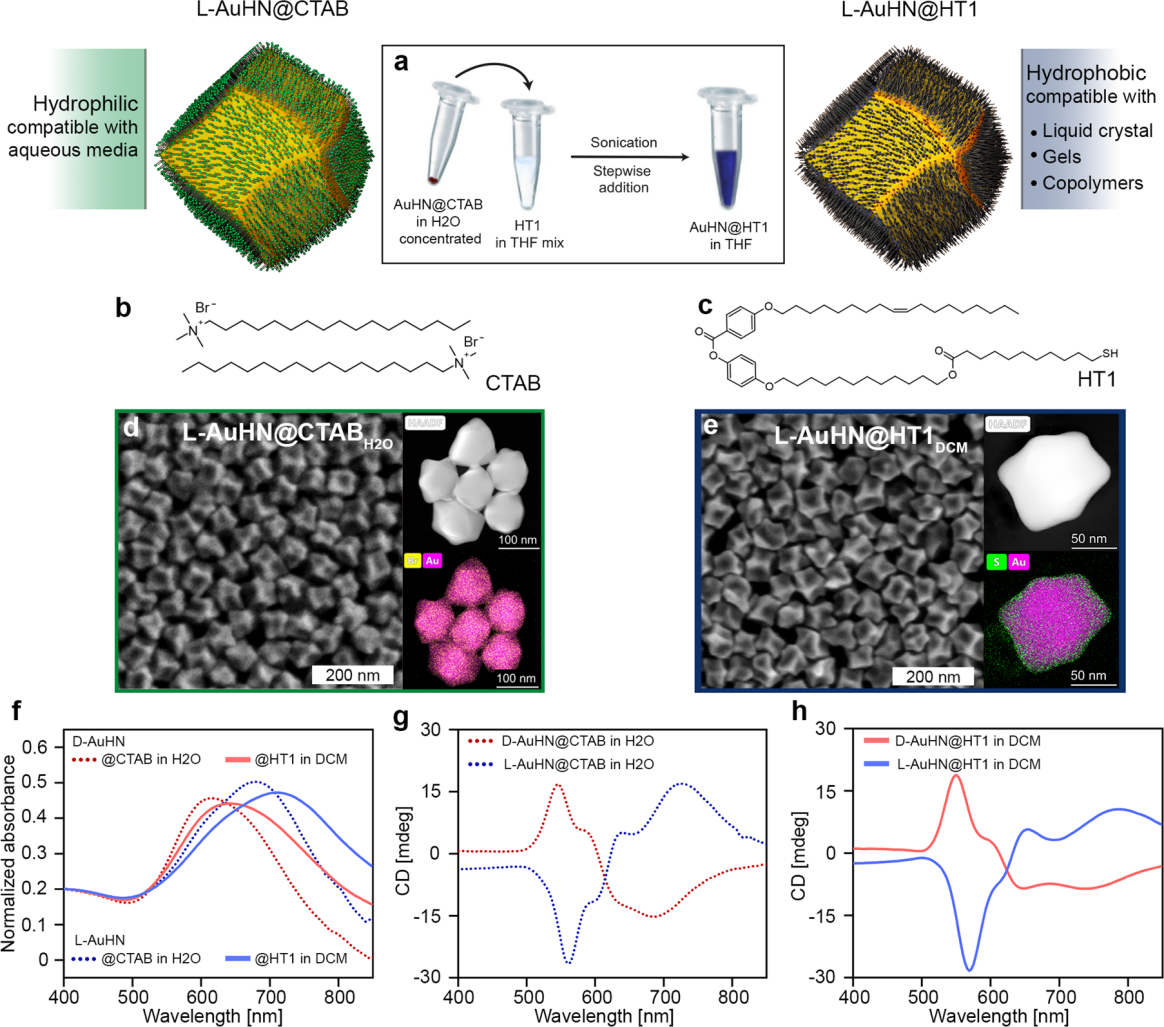
Outline of hydrophobic chiral plasmonic NPs fabrication. (a) Scheme of the phase transfer of l-AuHN NPs into the hydrophobic environment through CTAB to HT1 ligand-exchange reaction. Molecular structures of (b) CTAB and (c) HT1. Representative SEM, HAADF TEM, and elemental mapping images of (d) l-AuHN@CTAB NPs and (e) l-AuHN@HT1 NPs after phase transfer to dichloromethane (DCM). (f) Vis–NIR spectra of L-AuHN (blue) and d-AuHN (red) NPs dispersions in water and dichloromethane (dotted and solid lines, respectively). (g) CD spectra of l- and d-AuHN@CTAB NPs (blue and red dotted lines, respectively) in water. (h) CD spectra of l- and d-AuHN@HT1 NPs (blue and red solid lines, respectively) in DCM.

To achieve the intended goal, we decided to develop a two-step protocol relying on the synthesis of water dispersible chiral Au NPs and their phase transfer (Figure 1a–c). We argued that, if successful, the adopted approach should prove applicable to a wide variety of recently developed synthetic protocols for chiral plasmonic NPs, providing access to chiral products with tunable size, shape, and PCD properties. To develop the planned approach, cetyltrimethylammonium bromide (CTAB) stabilized helicoidal Au NPs were prepared using a modified literature protocol.^11^ Enantiomeric batches of these particles were prepared by overgrowing achiral, 50 nm cuboctahedron particles in the presence of a symmetry breaking inducer, either d- or l-cysteine. The shape of the obtained NPs resembled that of a rhombic dodecahedron of ∼110–120 nm as determined by SEM (Figure 1d and Figure S1 in the Supporting Information). We named these NPs l-AuHN@CTAB and d-AuHN@CTAB, which reflects: the use of l- or d-cysteine for morphology control, the helicoidal nanoparticle shape (AuHN), and CTAB coating, respectively.

Au NPs exhibited the localized surface plasmon resonance (LSPR) centered at ∼680 and ∼615 nm for l- and d-AuHN@CTAB, respectively (Figure 1f). CD spectra recorded for both enantiomeric NPs were almost mirror images, exhibiting Cotton characteristics, revealing the chiral nature of NPs. The first Cotton signal was centered at ∼560 and ∼545 nm and the second band at ∼725 and ∼680 nm, for l- and d-enantiomers, respectively (Figure 1g). The calculated dissymmetry factors (Note S1), g-factors, were on the order of ∼2–3 × 10^–3^.

To perform the phase transfer, we functionalized these chiral NPs with a hydrophobic, liquid crystal thiol 4-((12-((11-sulfanyloundecanoyl)oxy)dodecyl)oxy)phenyl-4-(octadec-9-en-1-yloxy)benzoate (HT1, Figure 1c). The molecular architecture of HT1 broadened interactions with solvents by benefiting from the presence of both alkyl and aromatic parts, similar to the strategy used in dendritic ligands design,^58−62^ while, due to a lower molecular weight, not producing a thick organic shell that could camouflage the chiral features of nanoparticles. Furthermore, alkyl chain flexibility, and the nonuniform surface of particles, can lead to mimicking a mixture of shorter and longer ligands in an organic shell, which was previously shown to be beneficial for colloidal stability of nonpolar nanoparticles.^57^ Thus, designing a HT1 structure comprising a stiff, aromatic core substituted with two alkyl chains seemed promising for the stabilization of relatively large NPs. Before phase transfer, l- and d-AuHN@CTAB NPs were concentrated to ∼50 mM and the CTAB concentration was kept between 0.5–1 mM, close to the critical micelle concentration (CMC). Then, the solution was added dropwise and under sonication to a tetrahydrofuran (THF) solution containing HT1 ligand in excess (1:1.4 NPs/HT1 molar ratio, Figure 1a). This methodology leads to a reduction of the surfactant concentration below the CMC, facilitating the diffusion and attachment of the thiol ligands due to the limitation of steric hindrance by CTAB molecules, similar to some of previously described ligand exchange protocols.^43,64,65^ After overnight incubation, the unbound HT1 was removed by centrifugation and the resulting HT1 functionalized Au NPs (l- and d-AuHN@HT1 NPs) were dispersed in a nonpolar organic solvent, dichloromethane (DCM). Successful exchange of ligands was further confirmed by evidencing the lack of bromine and the presence of sulfur atoms on the surface of phase transferred NPs using transmission electron microscopy (TEM) elemental mapping (Figure 1d,e).

l- and d-AuHN@HT1 NPs dispersions conserved colloidal stability in DCM. For these samples, well-defined LSPR bands were detected, centered at ∼710 and 640 nm for l- and d-AuHN@HT1, respectively. LSPR bands were red-shifted by ∼25–30 nm in comparison to AuHN@CTAB NPs water dispersions (Figure 1f) due to the changes in the local refractive index produced by the ligand functionalization and solvent exchange. Importantly, CD spectra of the phase transferred NPs preserved characteristic Cotton bands, showing only a slight shift from the starting material. For l- and d-AuHN@HT1 DCM dispersions, main bands were centered at ∼570 and ∼550 nm, respectively (Figure 1h). Furthermore, no significant changes in the g-factor values were observed. The values were ca. −3.2 × 10^–3^ and 2.2 × 10^–3^ for l- and d-AuHN@HT1 in DCM versus ca. −3.0 × 10^–3^ and 2.0 × 10^–3^ for l- and d-AuHN@CTAB in H_2_O samples, respectively (Figure S2). Finally, NPs conserved morphological integrity after ligand-exchange procedure as confirmed by SEM analysis (Figure 1d,e and Figure S3).

### Colloidal Stability in Hydrophobic Systems

**Figure 2:**
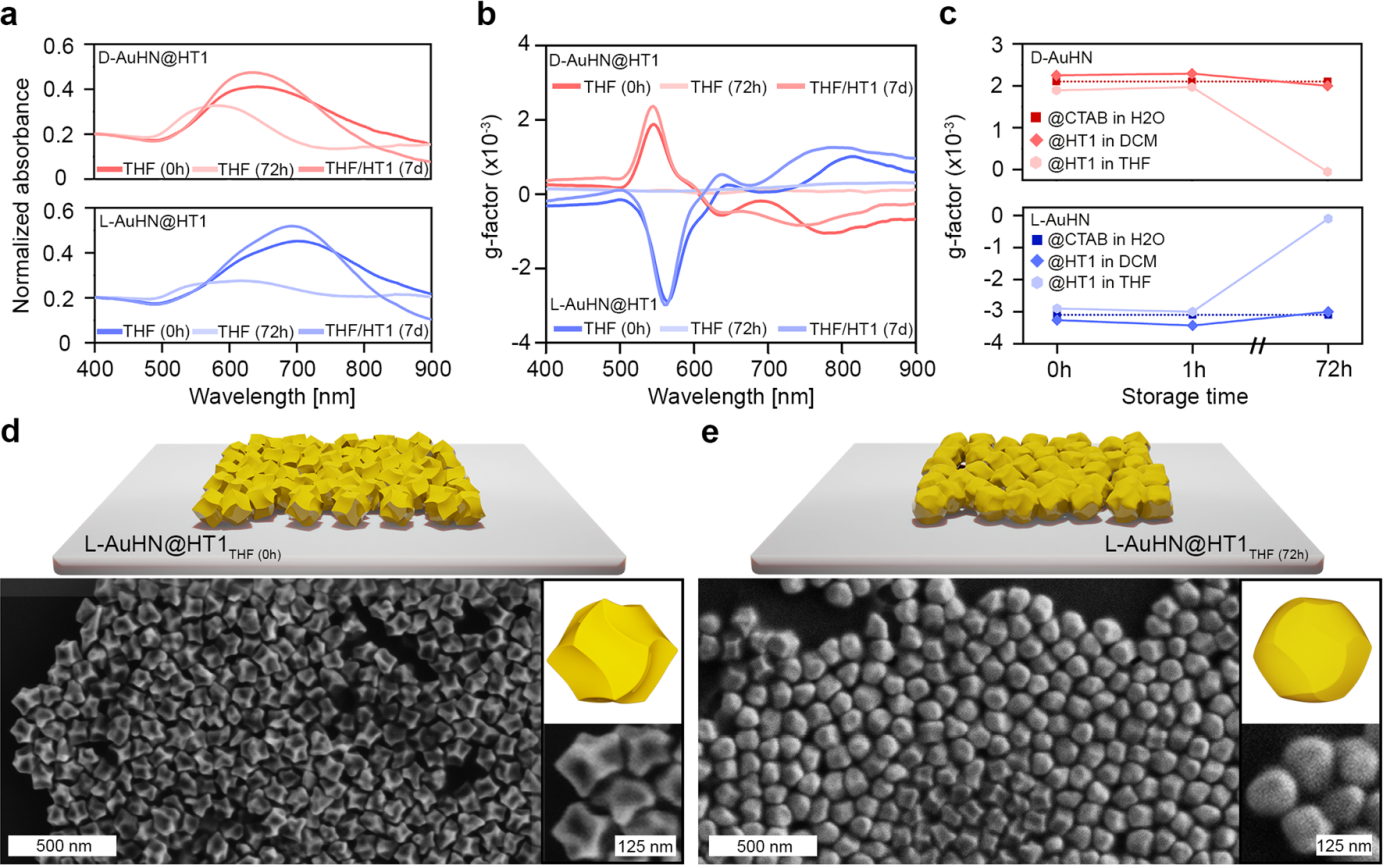
Stability of hydrophobic, chiral NPs. (a) Vis–NIR spectra of d- and l-AuHN@HT1 NPs (upper and lower spectra, respectively) dispersed in THF at various storage times: 0 h, 72 h without HT1 excess, and 7 days with HT1 excess. (b) CD spectra corresponding to those shown in a. (c) G-factor calculated at the peak of the main CD band shown in panel b, compared to that of AuHN@CTAB in H_2_O. (d and e) SEM micrographs and 3D models of dropcasted l-AuHN@HT1 NPs in THF (0 h) and l-AuHN@HT1 NPs in THF (72 h); insets present zoom into NPs.

We next tested the colloidal stability of l- and d-Au@HT1 NPs in various hydrophobic solvents commonly used for soft-matter composites fabrication. To evaluate the colloidal stability, the optical properties of these chiral NPs dispersed in toluene (TOL), THF, and DCM were monitored with time (0, 1, and 72 h). The optical properties of AuHN@HT1 NPs in DCM and TOL were almost identical and remained unaltered throughout 72 h, as attested by Vis–NIR and CD spectroscopies (Figure S2). Notably, g-factor values, calculated at the first Cotton band, varied less than 10%. These results suggest that characteristic helicoidal morphology and the surface coating of NPs were not affected throughout the tested period (Figure 2c and Figures S2 and S3a,b). In a clear contrast, AuHN@HT1 NPs in THF were much less stable (Figure 2a,b). After 72 h, we noted a significant decrease, broadening, and blue shift of the LSPR bands, and no CD bands were evidenced. SEM analysis of the samples directly after phase transfer and after storing in THF for 72 h concorded with the optical characterization and showed rounding of the nanoparticles (Figure 2d,e and Figure S3c,d). The effect of lower dispersibility and reshaping of NPs in THF could potentially be caused by higher polarity and water content or some impurities of THF in comparison to other solvents tested, leading to faster precipitation and ligand stripping. Moreover, we hypothesize that the observed effects could also be related to the exposure of high-Miller-index Au planes, characteristic for intrinsically chiral Au NPs.^16^ The presence of high-index facets could affect the interaction of ligands with the NP surface, lowering surface coverage and thus compromising the colloidal stability of intrinsically chiral NPs in comparison to more symmetric ones, exposing low-index planes. To test this hypothesis, we performed an analogous stability experiment for spherical NPs (AuNS) coated with hydrophobic thiol and stored in THF. The stability of AuNS dispersion was monitored using Vis–NIR spectroscopy. In agreement with the hypothesis, we observed a significantly smaller degrading effect of storing AuNS in THF than in the case of AuNH (Figure S4, Note S2). As the next step, we decided to experimentally confirm if the colloidal stability of the chiral AuHN@HT1 NPs in THF can be improved. We dispersed NPs in THF containing free HT1 ligands (5:1 Au@HT1 to free HT1 molar ratio), observing that the samples maintained their PCD properties almost unaltered for at least 7 days (Figure 2a,b). Overall, these results suggest that, for practical applications, hydrophobic chiral NPs should be stored in THF without removing HT1 excess; the purification step should be performed directly before using NP.

### Universality of the Phase Transfer Process

**Figure 3:**
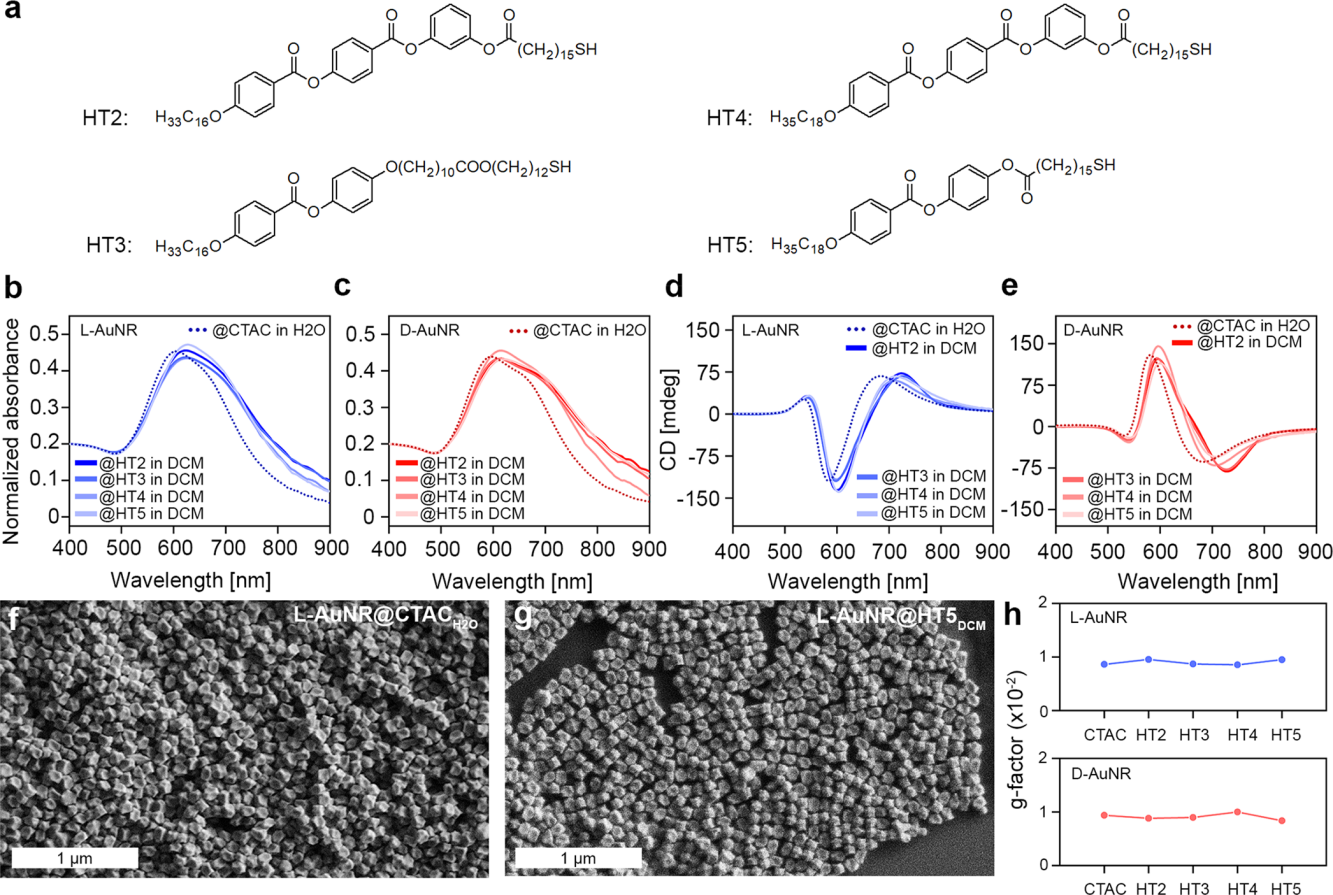
Universality of phase transfer of chiral nanoparticles via hydrophobic thiol ligands (HT). (a) Structures of HT used for the phase transfer process. (b and c) Vis–NIR spectra of l- and d-AuNRs dispersions in water (AuNR@CTAC in H_2_O) and dichloromethane (AuNR@HT2–5 in DCM). (d and e) Circular dichroism spectra of dispersions of hydrophilic and hydrophobic l-AuNR and d-AuNR. (f and g) Representative SEM micrographs of l-AuNR before (f) and after (g) ligand exchange, samples were dropcasted from water and dichloromethane dispersions, respectively. (h) Absolute value of g-factor calculated at the maximum of the main CD band for l-AuNR (at the top) and d-AuNR (bottom) coated with different hydrophobic thiols dispersed in DCM, compared to hydrophilic l- and d-AuNR coated with CTAC dispersed in water.

To confirm the universality of our phase transfer methodology, we tested it against using a different type of intrinsically chiral nanoparticles, CTAC stabilized gold nanorods (AuNRs@CTAC), and a set of liquid-crystal like ligands (HT2-HT5, Figure 3a). Toward this aim, the d- and l-handed chiral AuNRs (∼117 nm length, ∼80 nm width) dispersed in water (d- and l-AuNR@CTAC in H_2_O, respectively) were synthesized following a modified previously reported protocol (Figure 3f and Figures S5 and S6).^63^ Variation of the hydrophobic ligands design was planned to enable assessment the role of molecular parts that may be crucial in the phase transfer process, namely, alkyl spacer chain length and the number of aromatic rings. After the phase transfer, the main LSPR band centered at ∼605 and ∼595 nm for l- and d-AuNR@CTAC in H_2_O, respectively (Figure 3b,c), red-shifted by ∼15–20 nm for both enantiomers, regardless the thiol ligand, which we ascribe to the surface functionalization and the change in the refractive index of the solvent (Table S1). Importantly, CD spectroscopy confirmed the PCD properties of nanoparticles related to their chiral morphology both in the hydrophilic and hydrophobic environment (Figure 3d,e). The phase transfer caused a red shift of the first Cotton band by ∼12–20 nm (Table S2) and less than 10% change of the g-factor values calculated at the maxima of the first Cotton band for hydrophilic NPs (−0.86 × 10^–2^ and 0.94 × 10^–2^ for l- and d-AuNR@CTAC in H_2_O, respectively, Figure 3h, Table S3). To verify the effect of phase transfer on the morphology, AuNR@HT5, dispersed in THF and DCM and containing an excess of HT5, was dropcasted on solid substrate after 3 days of aging and analyzed by SEM. Indeed, the chiral shape of NPs was preserved in all tested samples (Figure 3g and Figures S7–S10). Overall, the optical spectroscopy and electron microscopy results proved the successful phase transfer of chiral AuNRs, attesting to the universality of the method toward different types of gold NPs with chiral morphology. Moreover, the fact that all tested HT ligands give similar stability leads to a conclusion that the exact design of stiff, aromatic core, and alkyl ligands is not the predominant factor in ensuring colloidal stability of NPs, and number of aromatic rings (2–3) and alkyl chain length (16–23 atoms in a chain) can be easily varied without affecting it. It also is worth noting that the methodology proposed here could be suitable for the transfer of NPs coated with relatively weakly bound surface ligands.

### Chiral Plasmonic Nanocomposites Fabrication

To test the applicability of hydrophobic chiral gold nanoparticles in soft matter composites formation, particularly in the context of their chemical compatibility to matrix and durability, we combined them with a set of three different hydrophobic, organic materials.

**Figure 4:**
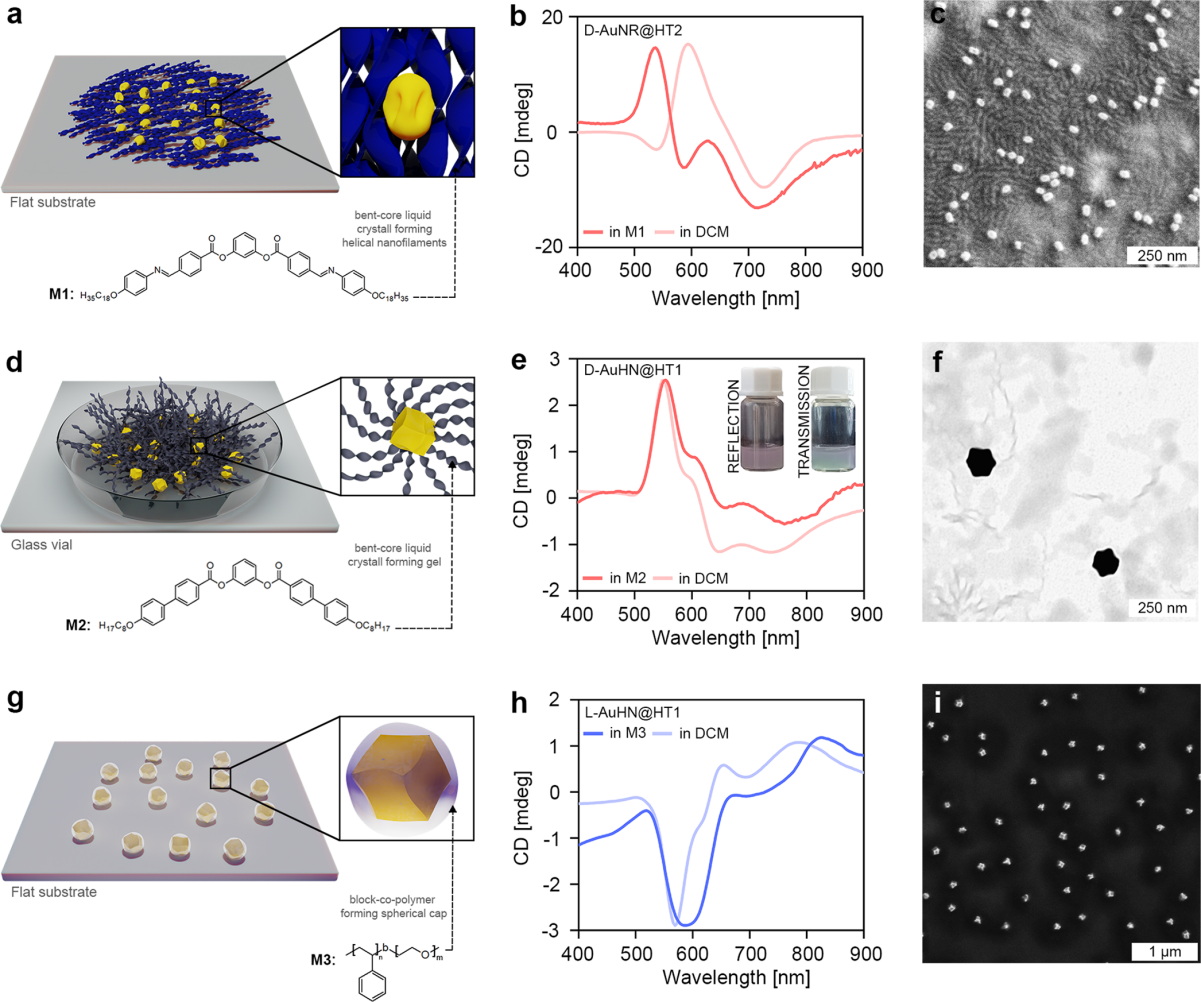
Compatibility of hydrophobic, chiral NPs with various organic materials serving as matrixes hosting chiral Au NPs. (a) Scheme of heat annealed d-AuNR@HT2 in M1 composite, comprising d-AuNR@HT2 NPs (shown in yellow) and M1 matrix (shown in blue), which is a liquid crystal forming helical nanofilaments in thin films. (b) Circular dichroism spectra of d-AuNR@HT2 in M1 sample after a heating–cooling cycle (155–30 °C) and d-AuNR@HT2 NPs dispersed in dichloromethane. (c) SEM micrograph of a heat annealed d-AuNR@HT2 in M1 sample. (d) Scheme of d-AuHN@HT1 in M2 gel composite, comprising d-AuHN@HT1 NPs (shown in yellow) and M2 matrix (shown in blue) forming physical gel by assembling into helical nanofilaments. (e) CD spectrum of d-AuHN@HT1 in M2 composite after gelation process and d-AuHN@HT1 NPs dispersed in dichloromethane; insets show optical photographs of the prepared samples in reflected and transmitted light. (f) TEM micrograph of d-AuHN@HT1 in M2 xerogel. (g) Scheme of l-AuHN@HT1 in M3 composite material comprising l-AuHN@HT1 NPs (yellow) and diblock copolymer (styrene blocked with polyethylene glycol, M3, bluish color) forming phase-separated spherical and cylindrical structures. (h) CD spectra of l-AuHN@HT1 in M3 composite dropcasted onto a solid substrate compared to CD spectra of AuHN@HT1 dispersion in DCM. (i) SEM micrograph of l-AuHN@HT1 in M3 composite.

The first was an oleyl-imine-matrix (M1), a liquid crystalline compound, which upon cooling from an elevated temperature (isotropic phase) forms an LC phase composed of helical nanofilaments (Figure 4a).^66^ Notably, the heat annealing procedure, required for achieving composites of helical nanofilaments with nanoinclusions, imposes requirements of thermal stability of NPs up to 155 °C. Therefore, it is important to investigate not only the chemical compatibility of AuNRs to M1 but also their thermal stability. A composite comprising M1 doped with d-AuNR@HT2 (1:2 Au^0^/M1 mass ratio), referred to as d-AuNR@HT2 in M1, was prepared and dropcasted onto a glass substrate and subjected to a heating–cooling cycle (heating to 155 °C and cooling to 30 °C). Vis–NIR analysis of the composites revealed a broad LSPR band with maxima at ∼560 nm, blue-shifted by ∼55 nm in comparison to the samples dispersed in DCM (Figure S11). CD spectra recorded after heat annealing confirmed the presence of PCD bands blue-shifted in comparison to a dispersed state (d-AuNR@HT2 in DCM, Figure 4b). The observed shift and broadening of the LSPR and PCD bands indicated possible plasmon coupling between NPs in the film state.

SEM imaging of the obtained d-AuNR@HT2 in M1 composites revealed the presence of well-dispersed d-AuNR@HT2 NPs solely in regions covered by M1, attesting to the chemical compatibility of d-AuNR@HT2 NPs with the M1 matrix (Figure 4c and Figure S12). Interestingly, SEM images also show that AuNRs preserve their morphology as well the presence of AuNR dimers, often with side-to-side geometry (Figure S12). These observations confirm our hypothesis that LSPR and PCD shift and broadening may be caused by partial plasmon coupling.^67,68^ Overall, the performed analyses of d-AuNR@HT2 in M1 composite attested that d-AuNR@HT2 NPs meet the requirement of chemical compatibility and high-temperature stability, needed for composite film preparation. Therefore, we conclude that embedding chiral, hydrophobic NPs within an organic matrix is a convenient way to increase their stability in the thin-film state (Figure S11).

Next, we attempted the formation of chiral composite gels comprising 1,3-phenylene bis-4-(4′-octadecyloxy)biphenyl) carboxylate (M2) and hydrophobic NPs. It was previously reported that M2 molecules form a supramolecular gel made of filaments, on cooling M2 solution in cyclohexane from 80 °C to room temperature (Figure 4d).^69^ Thus, to prepare the nanocomposite gel, a colloidal dispersion of ∼50 mM d-AuHN@HT1 NPs in DCM was added to 10 mg/mL M2 in cyclohexane (1:20 Au^0^/M2 mass ratio). After homogenization at 80 °C, the sample was left at ambient conditions for cooling. The resulting gel, named d-AuHN@HT1 in M2, exhibited weak LSPR and PCD properties, with bands position similar to those of d-AuHN@HT1 in DCM (Figure 4e and Figure S13). When observed in reflected or transmitted light, the gel showed the characteristic colors of d-AuHN NPs dispersion in liquid phase. TEM images of the sample revealed the presence of individual NPs in contact M2 gel filaments (Figure 4f and Figure S13). Overall, optical and structural investigations of d-AuHN@HT1 in M2 composite attested that d-AuHN@HT1 NPs exhibit compatibility with the M2 matrix and can be conveniently built into the supramolecular structure of the gel.

Finally, we tested the compatibility of hydrophobic, chiral NPs with poly(styrene)-*b*-poly(ethylene oxide) block copolymer molecules (PS-*b*-PEO, referred to as matrix M3, Figure 4g). Favorable interactions between hydrophobic NPs and the hydrophobic polystyrene component of PS-*b*-PEO were previously reported.^70^ The composite, l-AuHN@HT1 in M3, was prepared by mixing M3 solution (1 mg/mL) with a colloidal dispersion of 50 mM l-AuHN@HT1 in DCM NPs (5:1 Au^0^/M3 mass ratio). For Vis–NIR absorption and CD measurements, the sample was dropcasted onto a quartz glass and washed with ethanol. The dropcasted l-AuHN@HT1in M3 showed a very weak LSPR band, at wavelengths similar to those observed for l-AuHN@HT1 in DCM (Note S3 and Figures S14 and S15). CD spectra revealed the presence of Cotton bands with maxima positions matching those of l-AuHN@HT1 in DCM, indicating that NPs are uniformly distributed within the polymer matrix. SEM analysis further confirmed that NPs preserved chiral structural features. The composite comprises mostly well-separated, individual NPs surrounded by spherical and worm-like structures of organic material, M3 block copolymer (Figure 4i and Figure S14).

## Conclusions

In conclusion, we described a simple and versatile method to resolve the problem of the availability of hydrophobic, morphologically chiral plasmonic nanoparticles. The developed approach relies on well-established synthetic methods yielding morphologically chiral nanoparticles in aqueous dispersions, followed by a phase transfer process using low-molecular weight hydrophobic ligands. Notably, the developed protocol yields morphologically chiral gold nanoparticles that are easily dispersible in hydrophobic solvents without compromising their chiral morphology and chiral optical properties (PCD properties). We highlight the universality and reproducibility of this method by successful phase transfer of two types of chiral nanoparticles using five types of ligands. The benefits of achieving hydrophobic, chiral plasmonic NPs are exemplified by the fabrication of composites with hydrophobic soft materials. We show that liquid crystals, physical gels, and block copolymers can be efficiently combined with chiral nanoparticles leading to composites exhibiting PCD properties. The efficient and nondamaging phase transfer of intrinsically chiral NPs described here unlocks the possibility to construct soft-matter composites benefiting from chiral properties of nanoinclusions, e.g., composites responsive to chiral light, or composites exhibiting chirality transfer from NPs. This paves the way for the creation of functional composites interesting for chirality sensing, smart material, and chiral photonic applications.

## Materials and Methods

### Materials

Tetrachloroauric acid (HAuCl_4_ ≥ 99%), hexadecyltrimethylammonium chloride (CTAC, 25 wt % in water), hexadecyltrimethylammonium bromide (CTAB, ≥ 99%), sodium borohydride (NaBH_4_), l-ascorbic acid (AA, ≥ 99%), silver nitrate (AgNO_3_ ≥ 99%), hydrochloric acid 36.5–38%, tetrahydrofuran (THF, anhydrous, ≥ 99.9%), toluene (anhydrous, ≥ 99.8%), and dichloromethane (DCM, anhydrous, ≥ 99.8%) were purchased from Sigma-Aldrich. l- and d-Cysteine hydrochloride monohydrate (>99.0%) were purchased from TCI. Poly(styrene)-*b*-poly(ethylene oxide) block copolymer (PS-*b*-PEO, C192 kg/mol) was purchased from Polymer Source. All chemicals were used without further purification. Milli-Q water was used in all experiments.

### Synthesis of Cuboctahedral Seeds

The synthesis of cuboctahedral seeds was carried out according to the slightly modified seed-mediated growth method reported by Nam et al.^11,71^ To obtain the initial seed solution, 0.25 mL of 10 mM HAuCl_4_ was added to 7.5 mL of 100 mM CTAB. After 5 min of stirring 0.8 mL of freshly prepared and ice-cold 10 mM NaBH_4_ was added under vigorous stirring, and the mixture was left undisturbed for 3 h. Subsequently, a growth solution was prepared by adding 1.6 mL of 100 mM CTAB and 0.2 mL of 10 mM HAuCl_4_ into 8 mL of water. After 5 min of stirring, 0.95 mL of 50 mM AA was added to the mixture. Finally, 10 μL of the previously prepared seed solution was added to the growth solution, mixed, and left undisturbed for 30 min. After incubation, obtained nanoparticles were washed twice by centrifugation (5000 rpm over 5 min) and redispersed in 1 mM CTAB solution for further use.

### Synthesis of Chiral Gold Helicoidal Nanoparticles (AuHN@CTAB_H_2_O_)

The synthesis of AuHN@CTAB_H_2_O_ was carried out according to the seed-mediated growth method reported by Nam et al.,^11^ scaled up 20 times. Previously prepared cuboctahedral NPs dispersion was used as a seed solution. The growth solution was prepared by adding 16 mL of 100 mM CTAB and 2 mL of 10 mM HAuCl_4_ into 79 mL of water and stirring for 5 min. Then, 9.5 mL of 0.1 M AA, 2 mL of seed solution, and 100 μL of 0.1 mM l-Cys/d-Cys were added, respectively, and the mixture was left undisturbed for 2 h at 30 °C. The final solution was centrifuged twice (5000 rpm over 5 min) to remove unreacted reagents and redispersed in a 1 mM CTAB solution for storage and further characterization. We named the obtained NPs l-AuHN@CTAB and d-AuHN@CTAB, which correspond to using l- or d- cysteine for morphology control, the helicoidal shape of nanoparticles, and CTAB coating. The measured dissymmetry factors (g-factors) were slightly lower than in the original submission, which is not surprising given the large scale of the synthesis, but it does not compromising our research aims.^72^

### Synthesis of Gold Rod-Like Shape Seeds

The synthesis of AuNRs was carried out according to a slightly modified seed-mediated growth method.^72^ To obtain the initial seed solution, 9.75 mL of 100 mM CTAB was mixed with 0.25 mL of 10 mM HAuCl_4_. After 5 min of stirring, 0.6 mL of freshly prepared and ice-cold 10 mM NaBH_4_ was added under vigorous stirring. Then, the mixture was left undisturbed at 27 °C for 2 h. Subsequently, a growth solution was prepared by adding 2 mL of 10 mM HAuCl_4_ into 40 mL of 0.1 M CTAB. Then, after 5 min of stirring, 290 μL of 10 mM AgNO_3_, 320 μL of 0.1 M AA, and 230 μL of 1.0 M HCl were added to the solution. Finally, 6 μL of previously prepared seed solution was added to the growth solution, mixed, and left undisturbed at 27 °C for 6 h.

### Synthesis of Chiral Gold Nanorods AuNR@CTAC_H_2_O_

The synthesis of chiral AuNR@CTAC_H_2_O_ was carried out according to the seed-mediated growth method reported by Wong et al.^63^ with slightly modifications. The solution of AuNRs prepared previously was washed once and concentrated until a gold concentration of 5 mM. Then, the concentrated solution was stored and used as a seed solution. The growth solution was prepared by adding 10 μL of 10 mM HAuCl_4_ into 4 mL of 40 mM CTAC and stirring for 5 min. Then, 475 μL of 0.1 M AA, 60 μL of 10 μM l-Cys/d-Cys, and 20 μL of seed solution were added, respectively, and the mixture was left undisturbed at 40 °C for 90 min. We named the obtained NPs l-AuNR@CTAC and d-AuNR@CTAC, which correspond to using l- or d- cysteine for morphology control, the rod-like shape of nanoparticles, and CTAC coating.

### Synthesis of the Organic Compounds–Hydrophobic Thiols (HT) and Matrixes (M)

Hydrophobic thiols HT1, HT2, and HT5 (as well as all intermediate compounds) were obtained following procedures described in our previous works.^44,66,73^ Compound HT3 was obtained using a procedure analogous to the synthesis of the compound HT1 (cetyl alcohol was used in the place of oleyl alcohol in step VI, Figure S16, Note S4). Compound HT4 was obtained using a procedure analogous to the synthesis of the compound HT2 (oleyl alcohol was used in the place of cetyl alcohol in step VI, Figure S16, Note S4).

M1 and M2 matrixes were obtained according to procedures described in our previous works.^66,69^

### Phase Transfer of Au NPs with Intrinsic Chirality

The obtained aqueous dispersions of hydrophilic, chiral nanoparticles (AuHN@CTAB_H_2_O_, AuNR@CTAC_H_2_O_) were centrifuged at 5000 rpm for 5 min. The pellet was redispersed in selected HT solution in THF in an ultrasonic bath. In a typical process, the molar ratio of Au^0^ to HT was 1:4. In the usual procedure, the 1:4 molar ratio corresponds to 0.5 mg Au^0^ content and ca. 3 mg of HT1–5. The mixture was left under mild stirring overnight. Afterward, the excess of HT ligand was removed via centrifugation (5000 rpm, 5 min) and NP redispersion in a pure solvent. Precipitates containing hydrophobic nanoparticles (AuHN@HT1–5 and AuNR@HT1–5) were dispersed in different solvents (dichloromethane, tetrahydrofuran, and toluene) for further measurements. We note two elements crucial for the successful phase transfer and maximization of the stability of hydrophobic, chiral NPs: (1) the precipitate containing chiral AuHN@CTAB/AuNR@CTAC should be added to the HT solutions dropwise; (2) after the first centrifugation, the supernatant should be discarded precisely to minimize the presence of water. The functionalized nanoparticles were named AuHN@HT1–5 and AuNR@HT1–5, which, as mentioned above, correspond to the helicoidal or rod-like shape of nanoparticles, as well as hydrophobic thiol coating.

### Preparation of Composites

#### d-AuNR@HT2-M1

Ten microliters of M1 DCM solution (10 mg/mL) was mixed with 5 μL of d-AuNR@HT2_DCM_ dispersion concentrated to ∼50 mM (∼0.05 mg Au^0^). Subsequently, the mixture was dropcasted onto indium-tin-oxide (ITO) glass. The composite film was then heated to 155 °C and cooled to 30 °C at 3 °C/min using a Linkam heating stage.

#### d-AuHN@HT1-M2

Ten milligrams of M2 compound was dissolved in 1 mL of cyclohexane at 80 °C. Fifty microliters of d-AuHN@HT1_DCM_ dispersion concentrated to ∼50 mM (∼0.5 mg Au^0^) was added immediately to a heated M2 cyclohexane solution and left in ambient conditions for cooling. For CD and TEM measurements, small portions (10% of total mass) of the gel were transferred to the quartz glass and TEM grid, respectively, using a metallic spatula. The sample prepared for TEM measurements was left to dry to form a xerogel.

#### l-AuHN@HT1-M3

Twenty microliters of M3 THF solution (1 mg/mL) was added to 10 μL of l-AuHN@HT1_THF_ dispersion concentrated to ∼50 mM (∼0.1 mg Au^0^). For CD measurements, the mixture was dropcasted onto a quartz glass and, after drying, washed with a small portion of ethanol. For SEM imaging, an analogous sample was prepared on the silica substrate.

## Methods

Structural analysis of nanomaterials was performed using transmission electron microscopy: TEM model JEM-1400 (JEOL), available in the Nencki Institute of Experimental Biology, laboratory of electron microscopy, TEM model JEM-1011 (JEOL) equipped with a model EDS INCA analyzer (Oxford, UK), in the Electron Microscopy Platform, Mossakowski Medical Research Centre Polish Academy of Science Warsaw. The structural analysis of nanomaterials was performed also via scanning electron microscopy: ZEISS SIGMA VP (Zeiss), available at the faculty of geology at the University of Warsaw. Vis–NIR absorption spectra were collected using a GENESYS 50 UV–vis spectrophotometer (Thermo Fisher Scientific, Waltham, MA), available at the University of Warsaw. NPs were redispersed using ultrasounds each time before measurements as they slowly precipitate (this applies both to hydrophilic and hydrophobic NPs): Sonic-3 (Polsonic) with power 2 × 160 W for a frequency of 40 kHz. PCD measurements were performed using a Chirascan circular dichroism spectrometer by Applied PhotoPhysics, available at the University of Warsaw. Absorption and CD spectra were smoothed using a Savitzky–Golay filter.

## Supporting Information Available

Figures of SEM and TEM micrographs, CD and g-factor spectra, Vis–NIR spectra, statistical analysis of the length and width, thermal stability test, synthetic route for the preparation of (pro)mesogenic ligands and tables of UV–vis–NIR analysis, circular dichroism analysis, and dissymmetry coefficient value (g-factor) analysis (PDF)

## References

[ref1] Wang L.; Hasanzadeh Kafshgari M.; Meunier M. Optical Properties and Applications of Plasmonic-Metal Nanoparticles. Adv. Funct. Mater. 2020, 30, 2005400 10.1002/adfm.202005400.

[ref2] Mueller N. S.; Okamura Y.; Vieira B. G. M.; Juergensen S.; Lange H.; Barros E. B.; Schulz F.; Reich S. Deep Strong Light–Matter Coupling in Plasmonic Nanoparticle Crystals. Nature 2020, 583 (7818), 780–784. 10.1038/s41586-020-2508-1. 32728238

[ref3] Zhao X.; Yang L.; Guo J.; Xiao T.; Zhou Y.; Zhang Y.; Tu B.; Li T.; Grzybowski B. A.; Yan Y. Transistors and Logic Circuits Based on Metal Nanoparticles and Ionic Gradients. Nat. Electron. 2021, 4 (2), 109–115. 10.1038/s41928-020-00527-z.

[ref4] Xia Y.; Gilroy K. D.; Peng H. C.; Xia X. Seed-Mediated Growth of Colloidal Metal Nanocrystals.. Angew. Chemie - Int. Ed. 2017, 56 (1), 60–95. 10.1002/anie.201604731. 27966807

[ref5] Zheng G.; He J.; Kumar V.; Wang S.; Pastoriza-Santos I.; Pérez-Juste J.; Liz-Marzán L. M.; Wong K. Y. Discrete Metal Nanoparticles with Plasmonic Chirality. Chem. Soc. Rev. 2021, 50 (6), 3738–3754. 10.1039/C9CS00765B. 33586721

[ref6] Im S. W.; Ahn H.; Kim R. M.; Cho N. H.; Kim H.; Lim Y.; Lee H.; Nam K. T. Chiral Surface and Geometry of Metal Nanocrystals. Adv. Mater. 2020, 32, 1905758 10.1002/adma.201905758. 31834668

[ref7] Hananel U.; Ben-Moshe A.; Tal D.; Markovich G. Enantiomeric Control of Intrinsically Chiral Nanocrystals. Adv. Mater. 2020, 32, 1905594 10.1002/adma.201905594. 31782846

[ref8] Wang H.; Liu Y.; Yu J.; Luo Y.; Wang L.; Yang T.; Raktani B.; Lee H. Selectively Regulating the Chiral Morphology of Amino Acid-Assisted Chiral Gold Nanoparticles with Circularly Polarized Light. ACS Appl. Mater. Interfaces 2022, 14 (2), 3559–3567. 10.1021/acsami.1c22191. 34982532

[ref9] Lee H.-E.; Ahn H.-Y.; Mun J.; Lee Y. Y.; Kim M.; Cho N. H.; Chang K.; Kim W. S.; Rho J.; Nam K. T. Amino-Acid- and Peptide-Directed Synthesis of Chiral Plasmonic Gold Nanoparticles. Nature 2018, 556 (7701), 360–365. 10.1038/s41586-018-0034-1. 29670265

[ref10] Lee Y. Y.; Cho N. H.; Im S. W.; Lee H. E.; Ahn H. Y.; Nam K. T. Chiral 432 Helicoid II Nanoparticle Synthesized with Glutathione and Poly(T)20 Nucleotide. ChemNanoMat 2020, 6 (3), 362–367. 10.1002/cnma.201900709.

[ref11] Lee H. E.; Kim R. M.; Ahn H. Y.; Lee Y. Y.; Byun G. H.; Im S. W.; Mun J.; Rho J.; Nam K. T. Cysteine-Encoded Chirality Evolution in Plasmonic Rhombic Dodecahedral Gold Nanoparticles. Nat. Commun. 2020, 11 (1), 263 10.1038/s41467-019-14117-x. 31937767 PMC6959252

[ref12] Wang S.; Zheng L.; Chen W.; Ji L.; Zhang L.; Lu W.; Fang Z.; Guo F.; Qi L.; Liu M. Helically Grooved Gold Nanoarrows: Controlled Fabrication, Superhelix, and Transcribed Chiroptical Switching. CCS Chem. 2021, 3, 2473–2484. 10.31635/ccschem.020.202000472.

[ref13] Zhang N. N.; Sun H. R.; Xue Y.; Peng F.; Liu K. Tuning the Chiral Morphology of Gold Nanoparticles with Oligomeric Gold-Glutathione Complexes. J. Phys. Chem. C 2021, 125 (19), 10708–10715. 10.1021/acs.jpcc.1c01641.

[ref14] Shi B.; Qu A.; Wang W.; Lu M.; Xu Z.; Chen C.; Hao C.; Sun M.; Xu L.; Xu C.; Kuang H. Chiral Cu_x_Co_y_S Supraparticles Ameliorate Parkinson’s Disease. CCS Chem. 2022, 4, 2440–2451. 10.31635/ccschem.021.202101107.

[ref15] Wu F.; Tian Y.; Luan X.; Lv X.; Li F.; Xu G.; Niu W. Synthesis of Chiral Au Nanocrystals with Precise Homochiral Facets for Enantioselective Surface Chemistry. Nano Lett. 2022, 22 (7), 2915–2922. 10.1021/acs.nanolett.2c00094. 35362992

[ref16] Cho N. H.; Byun G. H.; Lim Y.; Im S. W.; Kim H.; Lee H.; Ahn H.; Nam K. T. Uniform Chiral Gap Synthesis for High Dissymmetry Factor in Single Plasmonic Gold Nanoparticle. ACS Nano 2020, 14 (3), 3595–3602. 10.1021/acsnano.9b10094. 32134639

[ref17] Jiang W.; Qu Z. B.; Kumar P.; Vecchio D.; Wang Y.; Ma Y.; Bahng J. H.; Bernardino K.; Gomes W. R.; Colombari F. M.; Lozada-Blanco A.; Veksler M.; Marino E.; Simon A.; Murray C.; Muniz S. R.; de Moura A. F.; Kotov N. A. Emergence of Complexity in Hierarchically Organized Chiral Particles. Science 2020, 368 (6491), 642–648. 10.1126/science.aaz7949. 32273399

[ref18] Yeom J.; Santos U. S.; Chekini M.; Cha M.; De Moura A. F.; Kotov N. A. Chiromagnetic Nanoparticles and Gels. Science 2018, 359 (6373), 309–314. 10.1126/science.aao7172. 29348234

[ref19] Hao C.; Xu L.; Sun M.; Ma W.; Kuang H.; Xu C. Chirality on Hierarchical Self-Assembly of Au@AuAg Yolk-Shell Nanorods into Core-Satellite Superstructures for Biosensing in Human Cells. Adv. Funct. Mater. 2018, 28 (33), 1802372 10.1002/adfm.201802372.

[ref20] Paiva-Marques W. A.; Gómez F. R.; Oliveira O. N.; Mejía-Salazar J. R. Chiral Plasmonics and Their Potential for Point-of-Care Biosensing Applications. Sensors 2020, 20 (3), 944 10.3390/s20030944. PMC7039232 32050725

[ref21] Wang G.; Hao C.; Ma W.; Qu A.; Chen C.; Xu J.; Xu C.; Kuang H.; Xu L. Chiral Plasmonic Triangular Nanorings with SERS Activity for Ultrasensitive Detection of Amyloid Proteins in Alzheimer’s Disease. Adv. Mater. 2021, 33, 2102337 10.1002/adma.202102337. 34309088

[ref22] Gwak J.; Park S. J.; Choi H. Y.; Lee J. H.; Jeong K. J.; Lee D.; Tran V. T.; Son K. S.; Lee J. Plasmonic Enhancement of Chiroptical Property in Enantiomers Using a Helical Array of Magnetoplasmonic Nanoparticles for Ultrasensitive Chiral Recognition. ACS Appl. Mater. Interfaces 2021, 13 (39), 46886–46893. 10.1021/acsami.1c14047. 34570473

[ref23] Ma Y.; Cao Z.; Hao J.; Zhou J.; Yang Z.; Yang Y.; Wei J. Controlled Synthesis of Au Chiral Propellers from Seeded Growth of Au Nanoplates for Chiral Differentiation of Biomolecules. J. Phys. Chem. C 2020, 124 (44), 24306–24314. 10.1021/acs.jpcc.0c07046.

[ref24] Khorashad L. K.; Besteiro L. V.; Correa-Duarte M. A.; Burger S.; Wang Z. M.; Govorov A. O. Hot Electrons Generated in Chiral Plasmonic Nanocrystals as a Mechanism for Surface Photochemistry and Chiral Growth. J. Am. Chem. Soc. 2020, 142 (9), 4193–4205. 10.1021/jacs.9b11124. 32026688

[ref25] Rafiei Miandashti A.; Khosravi Khorashad L.; Kordesch M. E.; Govorov A. O.; Richardson H. H. Experimental and Theoretical Observation of Photothermal Chirality in Gold Nanoparticle Helicoids. ACS Nano 2020, 14 (4), 4188–4195. 10.1021/acsnano.9b09062. 32176469

[ref26] Xu L.; Wang X.; Wang W.; Sun M.; Choi W. J.; Kim J. Y.; Hao C.; Li S.; Qu A.; Lu M.; Wu X.; Colombari F. M.; Gomes W. R.; Blanco A. L.; de Moura A. F.; Guo X.; Kuang H.; Kotov N. A.; Xu C. Enantiomer-Dependent Immunological Response to Chiral Nanoparticles. Nature 2022, 601 (7893), 366–373. 10.1038/s41586-021-04243-2. 35046606

[ref27] Zhang N. N.; Sun H. R.; Liu S.; Xing Y. C.; Lu J.; Peng F.; Han C. L.; Wei Z.; Sun T.; Yang B.; Liu K. Gold Nanoparticle Enantiomers and Their Chiral-Morphology Dependence of Cellular Uptake. CCS Chem. 2022, 4 (2), 660–670. 10.31635/ccschem.021.202000637.

[ref28] Hao C.; Qu A.; Xu L.; Sun M.; Zhang H.; Xu C.; Kuang H. Chiral Molecule-Mediated Porous Cu_x_O Nanoparticle Clusters with Antioxidation Activity for Ameliorating Parkinson’s Disease. J. Am. Chem. Soc. 2019, 141 (2), 1091–1099. 10.1021/jacs.8b11856. 30540450

[ref29] Hao C.; Wu X.; Sun M.; Zhang H.; Yuan A.; Xu L.; Xu C.; Kuang H. Chiral Core-Shell Upconversion Nanoparticle@MOF Nanoassemblies for Quantification and Bioimaging of Reactive Oxygen Species in Vivo. J. Am. Chem. Soc. 2019, 141 (49), 19373–19378. 10.1021/jacs.9b09360. 31711292

[ref30] Shukla N.; Gellman A. J. Chiral Metal Surfaces for Enantioselective Processes. Nat. Mater. 2020, 19 (9), 939–945. 10.1038/s41563-020-0734-4. 32747699

[ref31] Hwang M.; Yeom B. Fabrication of Chiral Materials in Nano- And Microscale †. Chem. Mater. 2021, 33 (3), 807–817. 10.1021/acs.chemmater.0c03846.

[ref32] Huang Y.; Nguyen M. K.; Natarajan A. K.; Nguyen V. H.; Kuzyk A. A DNA Origami-Based Chiral Plasmonic Sensing Device. ACS Appl. Mater. Interfaces 2018, 10 (51), 44221–44225. 10.1021/acsami.8b19153. 30525378

[ref33] Kim H.; Im S. W.; Cho N. H.; Seo D. H.; Kim R. M.; Lim Y. C.; Lee H. E.; Ahn H. Y.; Nam K. T. γ-Glutamylcysteine- and Cysteinylglycine-Directed Growth of Chiral Gold Nanoparticles and Their Crystallographic Analysis.. Angew. Chemie - Int. Ed. 2020, 59 (31), 12976–12983. 10.1002/anie.202003760. 32337812

[ref34] Luo J.; Cheng Y.; Gong Z. W.; Wu K.; Zhou Y.; Chen H. X.; Gauthier M.; Cheng Y. Z.; Liang J.; Zou T. Self-Assembled Peptide Functionalized Gold Nanopolyhedrons with Excellent Chiral Optical Properties. Langmuir 2020, 36 (2), 600–608. 10.1021/acs.langmuir.9b03366. 31885276

[ref35] Kim H.; Kim R. M.; Namgung S. D.; Cho N. H.; Son J. B.; Bang K.; Choi M.; Kim S. K.; Nam K. T.; Lee J. W.; Oh J. H. Ultrasensitive Near-Infrared Circularly Polarized Light Detection Using 3D Perovskite Embedded with Chiral Plasmonic Nanoparticles. Adv. Sci. 2022, 9, 2104598 10.1002/advs.202104598. PMC8844506 34978155

[ref36] Horrer A.; Zhang Y.; Gérard D.; Béal J.; Kociak M.; Plain J.; Bachelot R. Local Optical Chirality Induced by Near-Field Mode Interference in Achiral Plasmonic Metamolecules. Nano Lett. 2020, 20 (1), 509–516. 10.1021/acs.nanolett.9b04247. 31816242

[ref37] Probst P. T.; Mayer M.; Gupta V.; Steiner A. M.; Zhou Z.; Auernhammer G. K.; König T. A. F.; Fery A. Mechano-Tunable Chiral Metasurfaces via Colloidal Assembly. Nat. Mater. 2021, 20 (7), 1024–1028. 10.1038/s41563-021-00991-8. 33927391

[ref38] Wu W.; Pauly M. Chiral Plasmonic Nanostructures: Recent Advances in Their Synthesis and Applications. Mater. Adv. 2022, 3 (1), 186–215. 10.1039/D1MA00915J.

[ref39] Perera K.; Nemati A.; Mann E. K.; Hegmann T.; Jákli A. Converging Microlens Array Using Nematic Liquid Crystals Doped with Chiral Nanoparticles. ACS Appl. Mater. Interfaces 2021, 13 (3), 4574–4582. 10.1021/acsami.0c21044. 33411492

[ref40] Morisawa K.; Ishida T.; Tatsuma T. Photoinduced Chirality Switching of Metal-Inorganic Plasmonic Nanostructures. ACS Nano 2020, 14 (3), 3603–3609. 10.1021/acsnano.9b10216. 32159939

[ref41] Pastoriza-Santos I.; Kinnear C.; Pérez-Juste J.; Mulvaney P.; Liz-Marzán L. M. Plasmonic Polymer Nanocomposites. Nat. Rev. Mater. 2018, 3 (10), 375–391. 10.1038/s41578-018-0050-7.

[ref42] Vila-Liarte D.; Kotov N. A.; Liz-Marzán L. M. Template-Assisted Self-Assembly of Achiral Plasmonic Nanoparticles into Chiral Structures. Chem. Sci. 2022, 13, 595–610. 10.1039/D1SC03327A. 35173926 PMC8768870

[ref43] Lewandowski W.; Szustakiewicz P.; Kowalska N.; Grzelak D.; Narushima T.; Góra M.; Bagiński M.; Pociecha D.; Okamoto H.; Liz-Marzán L. M. Supramolecular Chirality Synchronization in Thin Films of Plasmonic Nanocomposites. ACS Nano 2020, 14 (10), 12918–12928. 10.1021/acsnano.0c03964. 32886482 PMC7596782

[ref44] Bagiński M.; Tupikowska M.; González-Rubio G.; Wójcik M.; Lewandowski W. Shaping Liquid Crystals with Gold Nanoparticles: Helical Assemblies with Tunable and Hierarchical Structures Via Thin-Film Cooperative Interactions. Adv. Mater. 2020, 32, 1904581 10.1002/adma.201904581. 31729083

[ref45] Nishikawa H.; Sano K.; Araoka F. Anisotropic fluid with phototunable dielectric permittivity. Nat. Commun. 2022, 13, 1142 10.1038/s41467-022-28763-1. 35241651 PMC8894468

[ref46] Jin X.; Jiang J.; Liu M. Reversible Plasmonic Circular Dichroism via Hybrid Supramolecular Gelation of Achiral Gold Nanorods. ACS Nano 2016, 10 (12), 11179–11186. 10.1021/acsnano.6b06233. 28024330

[ref47] Yu H.; Welch C.; Qu W.; Schubert C. J.; Liu F.; Siligardi G.; Mehl G. H. Chirality Enhancement in Macro-Chiral Liquid Crystal Nanoparticles. Mater. Horizons 2020, 7 (11), 3021–3027. 10.1039/D0MH01274B.

[ref48] Nemati A.; Shadpour S.; Querciagrossa L.; Li L.; Mori T.; Gao M.; Zannoni C.; Hegmann T. Chirality Amplification by Desymmetrization of Chiral Ligand-Capped Nanoparticles to Nanorods Quantified in Soft Condensed Matter. Nat. Commun. 2018, 9 (1), 3908 10.1038/s41467-018-06400-0. 30254259 PMC6156227

[ref49] Heuer-Jungemann A.; Feliu N.; Bakaimi I.; Hamaly M.; Alkilany A.; Chakraborty I.; Masood A.; Casula M. F.; Kostopoulou A.; Oh E.; Susumu K.; Stewart M. H.; Medintz I. L.; Stratakis E.; Parak W. J.; Kanaras A. G. The Role of Ligands in the Chemical Synthesis and Applications of Inorganic Nanoparticles. Chem. Rev. 2019, 119 (8), 4819–4880. 10.1021/acs.chemrev.8b00733. 30920815

[ref50] Paramasivam G.; Kayambu N.; Rabel A. M.; Sundramoorthy A. K.; Sundaramurthy A. Anisotropic Noble Metal Nanoparticles: Synthesis, Surface Functionalization and Applications in Biosensing, Bioimaging, Drug Delivery and Theranostics. Acta Biomater. 2017, 49, 45–65. 10.1016/j.actbio.2016.11.066. 27915023

[ref51] Serrano-Montes A. B.; De Aberasturi D. J.; Langer J.; Giner-Casares J. J.; Scarabelli L.; Herrero A.; Liz-Marzán L. M. A General Method for Solvent Exchange of Plasmonic Nanoparticles and Self-Assembly into SERS-Active Monolayers. Langmuir 2015, 31 (33), 9205–9213. 10.1021/acs.langmuir.5b01838. 26258732 PMC4550895

[ref52] Lista M.; Liu D. Z.; Mulvaney P. Phase Transfer of Noble Metal Nanoparticles to Organic Solvents. Langmuir 2014, 30 (8), 1932–1938. 10.1021/la404569h. 24479856

[ref53] Yang J.; Lee J. Y.; Ying J. Y. Phase Transfer and Its Applications in Nanotechnology. Chem. Soc. Rev. 2011, 40 (3), 1672–1696. 10.1039/B916790K. 21120233

[ref54] Hühn J.; Carrillo-Carrion C.; Soliman M. G.; Pfeiffer C.; Valdeperez D.; Masood A.; Chakraborty I.; Zhu L.; Gallego M.; Yue Z.; Carril M.; Feliu N.; Escudero A.; Alkilany A. M.; Pelaz B.; Pino P. Del; Parak W. J. Selected Standard Protocols for the Synthesis, Phase Transfer, and Characterization of Inorganic Colloidal Nanoparticles. Chem. Mater. 2017, 29 (1), 399–461. 10.1021/acs.chemmater.6b04738.

[ref55] Sánchez-Iglesias A.; Claes N.; Solís D. M.; Taboada J. M.; Bals S.; Liz-Marzán L. M.; Grzelczak M. Reversible Clustering of Gold Nanoparticles under Confinement. Angew. Chem., Int. Ed. 2018, 57, 3183 10.1002/anie.201800736. PMC6468316 29417726

[ref56] Yang Y.; Qin H.; Jiang M.; Lin L.; Fu T.; Dai X.; Zhang Z.; Niu Y.; Cao H.; Jin Y.; Zhao F.; Peng X. Entropic Ligands for Nanocrystals: From Unexpected Solution Properties to Outstanding Processability. Nano Lett. 2016, 16 (4), 2133–2138. 10.1021/acs.nanolett.6b00730. 26923682

[ref57] Pang Z.; Zhang J.; Cao W.; Kong X.; Peng X. Partitioning Surface Ligands on Nanocrystals for Maximal Solubility. Nat. Commun. 2019, 10 (1), 2454 10.1038/s41467-019-10389-5. 31165734 PMC6549164

[ref58] Elbert K. C.; Jishkariani D.; Wu Y.; Lee J. D.; Donnio B.; Murray C. B. Design, Self-Assembly, and Switchable Wettability in Hydrophobic, Hydrophilic, and Janus Dendritic Ligand-Gold Nanoparticle Hybrid Materials. Chem. Mater. 2017, 29 (20), 8737–8746. 10.1021/acs.chemmater.7b02928.

[ref59] Malassis L.; Jishkariani D.; Murray C. B.; Donnio B. Dendronization-Induced Phase-Transfer, Stabilization and Self-Assembly of Large Colloidal Au Nanoparticles. Nanoscale 2016, 8 (27), 13192–13198. 10.1039/C6NR03404G. 27348477

[ref60] Jishkariani D.; Wu Y.; Wang D.; Liu Y.; Van Blaaderen A.; Murray C. B. Preparation and Self-Assembly of Dendronized Janus Fe_3_O_4_-Pt and Fe_3_O_4_-Au Heterodimers. ACS Nano 2017, 11 (8), 7958–7966. 10.1021/acsnano.7b02485. 28771319

[ref61] Jishkariani D.; Diroll B. T.; Cargnello M.; Klein D. R.; Hough L. A.; Murray C. B.; Donnio B. Dendron-Mediated Engineering of Interparticle Separation and Self-Assembly in Dendronized Gold Nanoparticles Superlattices. J. Am. Chem. Soc. 2015, 137 (33), 10728–10734. 10.1021/jacs.5b06306. 26258660

[ref62] Elbert K. C.; Vo T.; Krook N. M.; Zygmunt W.; Park J.; Yager K. G.; Composto R. J.; Glotzer S. C.; Murray C. B. Dendrimer Ligand Directed Nanoplate Assembly. ACS Nano 2019, 13 (12), 14241–14251. 10.1021/acsnano.9b07348. 31756073

[ref63] Zheng G.; Bao Z.; Pérez-Juste J.; Du R.; Liu W.; Dai J.; Zhang W.; Lee L. Y. S.; Wong K. Y. Tuning the Morphology and Chiroptical Properties of Discrete Gold Nanorods with Amino Acids.. Angew. Chemie - Int. Ed. 2018, 57 (50), 16452–16457. 10.1002/anie.201810693. 30375752

[ref64] Indrasekara A. S. D. S.; Wadams R. C.; Fabris L. Ligand Exchange on Gold Nanorods: Going Back to the Future. Part. Part. Syst. Charact. 2014, 31 (8), 819–838. 10.1002/ppsc.201400006.

[ref65] Dewi M. R.; Laufersky G.; Nann T. A Highly Efficient Ligand Exchange Reaction on Gold Nanoparticles: Preserving Their Size, Shape and Colloidal Stability. RSC Adv. 2014, 4 (64), 34217–34220. 10.1039/C4RA05035E.

[ref66] Grzelak D.; Tupikowska M.; Vila-Liarte D.; Beutel D.; Bagiński M.; Parzyszek S.; Góra M.; Rockstuhl C.; Liz-Marzán L. M.; Lewandowski W. Liquid Crystal Templated Chiral Plasmonic Films with Dynamic Tunability and Moldability. Adv. Funct. Mater. 2022, 32, 2111280 10.1002/adfm.202111280.

[ref67] Szustakiewicz P.; Kowalska N.; Bagiński M.; Lewandowski W. Active Plasmonics with Responsive, Binary Assemblies of Gold Nanorods and Nanospheres. Nanomaterials 2021, 11 (9), 2296 10.3390/nano11092296. 34578613 PMC8465109

[ref68] Grzelak D.; Szustakiewicz P.; Tollan C.; Raj S.; Král P.; Lewandowski W.; Liz-Marzán L. M. In Situ Tracking of Colloidally Stable and Ordered Assemblies of Gold Nanorods. J. Am. Chem. Soc. 2020, 142 (44), 18814–18825. 10.1021/jacs.0c06446. 32990433 PMC7645924

[ref69] Matraszek J.; Topnani N.; Vaupotič N.; Takezoe H.; Mieczkowski J.; Pociecha D.; Gorecka E. Monolayer Filaments versus Multilayer Stacking of Bent-Core Molecules. Angew. Chem., Int. Ed. 2016, 55, 3468 10.1002/anie.201510123. 26833945

[ref70] Kirschner J.; Will J.; Rejek T. J.; Portilla L.; Berlinghof M.; Schweizer P.; Spiecker E.; Steinrück H. G.; Unruh T.; Halik M. Memory Effect of Self-Assembled PS-b-PEO Block Copolymer Films with Selectively Embedded Functionalized TiO2 Nanoparticles. Adv. Mater. Interfaces 2017, 4, 1700230 10.1002/admi.201700230.

[ref71] Ahn H. Y.; Lee H. E.; Jin K.; Nam K. T. Extended Gold Nano-Morphology Diagram: Synthesis of Rhombic Dodecahedra Using CTAB and Ascorbic Acid. J. Mater. Chem. C 2013, 1 (41), 6861–6868. 10.1039/c3tc31135j.

[ref72] Scarabelli L.; Sánchez-Iglesias A.; Pérez-Juste J.; Liz-Marzán L. M. A “Tips and Tricks” Practical Guide to the Synthesis of Gold Nanorods. J. Phys. Chem. Lett. 2015, 6 (21), 4270–4279. 10.1021/acs.jpclett.5b02123. 26538043

[ref73] Lewandowski W.; Fruhnert M.; Mieczkowski J.; Rockstuhl C.; Gorecka E. Dynamically self-assembled silver nanoparticles as a thermally tunable metamaterial. Nat. Commun. 2015, 6, 6590 10.1038/ncomms7590. 25779822

