# Supplementary material for: Hydrophobic Gold Nanoparticles with Intrinsic Chirality for the Efficient Fabrication of Chiral Plasmonic Nanocomposites
Source: ACS Appl Mater Interfaces. 2022 Oct 28;14(44):50013–23. doi: 10.1021/acsami.2c11925 (PMC9650650; doi:10.1021/acsami.2c11925)
Supplement: Supplementary file 1 — am2c11925_si_001.pdf [file am2c11925_si_001.pdf]

Supporting Information

Hydrophobic gold nanoparticles with intrinsic chirality for the efficient fabrication of chiral plasmonic nanocomposites

Natalia Kowalska[a], Filip Bandalewicz[a], Jakub Kowalski[a], Sergio Gómez-Graña[b,c], Maciej Bagiński[a], Isabel Pastoriza-Santos[b,c], Marek Grzelczak[d], Joanna Matrzaszek[a], Jorge Pérez-Juste[b,c], Wiktor Lewandowski[a]

a Laboratory of Organic Nanomaterials and Biomolecules, Faculty of Chemistry University of Warsaw, Pasteura 1 Street, 02-093 Warsaw, Poland

E-mail: wlewandowski@chem.uw.edu.pl

b CINBIO, Universidade de Vigo, Departamento de Química Física, Campus Universitario As Lagoas, Marcosende, 36310 Vigo, Spain.

c Instituto de Investigación Sanitaria Galicia Sur (IIS Galicia Sur), SERGAS-UVIGO, 36213 Vigo, Spain

d Centro de Física de Materiales (CSIC-UPV/EHU) and Donostia International Physics Center, 20018 Donostia – San Sebastián, Spain

Supplementary Note 1.

To estimate the optical activity, the dimensionless Kuhn factor (g-factor), which is a parameter describing the ratio of polarized photons to extinct ones, was used.[1] The g-factor values shown in Figures 2-3, Figures S2, and Table S3 were calculated using the following equation:

$$g_{abs} = \frac{\Delta A}{A}$$

where: ΔA – difference in absorbance between left- and right-handed circularly polarized light, A – total absorbance of the material

Supplementary Note 2.

Gold crystallizes in FCC symmetry, thus, achieving intrinsically chiral gold NPs with twisted surfaces translates to the fabrication of NPs with exposed high-index planes. The presence of high index facets, and high curvature of these entities, could affect the interaction of ligands with the NP surface leading to a less densely packed ligand shell, compromising the colloidal stability of chiral NPs vs. these more symmetric ones.

To verify this hypothesis we performed phase transfer of intrinsically chiral NPs (L-AuHN@CTAB, note that this is another batch of NPs than that reported in the main text, translating to minute differences in the optical properties) and spherical NPs (AuNS@CTAC). For this purpose, we used a hydrophobic thiol, HT5, which is similar to HT1 (see Figure 3 in the main text). Particles were stored in THF through 72 hours, without excess of HT5.

The stability of L-AuHN@HT5 and AuNS@HT5 was monitored using CD and UV-Vis-NIR spectroscopies. We found that in these conditions, AuHN@HT5 degraded over time, that is these particles exhibited the same tendency as AuHN@HT1 stored in the same conditions. Namely, we observed: (1) blueshift of the LSPR band, (2) decreasing of the CD bands after 72 hours, and (3)

rounding of NPs as probed with SEM. Although this process was less pronounced than in the experiment showcased in Figure 2, the noted tendencies were clear.

In the case of spherical nanoparticles (AuNS), which do not have high index planes, phase transfer using HT ligand resulted in a minute redshift of the LSPR band, associated with a change in refractive index, while decrease in absorbance was almost negligible. Overall, these results support the hypothesis that high index planes are less stable, affecting the colloidal stability of non-polar chiral nanoparticles

Supplementary Note 3.

The strength of CD response of composites deserves a broadened discussion.

A) Factors limiting the intensity of CD bands of composites:

In general, if chiral particles are not affected by the formation mechanism of composites, the registered CD signals should be comparable to those of NP dispersions Factors responsible for observed changes could be related to temperature-induced reshaping and clustering of NPs. Using TEM/SEM we confirmed structural changes of particles and clustering do not dominate the sample. To further investigate the matter, we calculated that in the case of D-AuNR@HT2 in M1 composite, the g-factor value diminished from 9*10^-3 to 3*10^-3. In the case of D-AuHN@HT1 in M2 and L-AuHN@HT1 in M3 composites, this difference was larger (2-3*10^-3 to 2-5*10-4) which indicates that either they cluster more, or simply these particles are less stable.

B) How to increase the registered CD response of composites:

In general, a few actions could be taken to increase the registered CD response of composites: increasing the thickness of probed composites, increasing the amount of added particles, or using intrinsically chiral NPs with stronger dissymmetry.

In this context, it is worth mentioning that, the field of intrinsically chiral NPs is rapidly growing, and new synthetic methods are published almost every month. For example, recently, by varying seeds (e.g. using triangular prisms) and chirality inductor (e.g. cysteine-phenylalanine dipeptide) a 10-100 fold increase in the chiroptical properties can be expected in comparison to the particle we were using. Thus, we are convinced tuning the wavelength range and increasing the intensity of the registered CD signals is further possible.

C) A proof-of-principle experiment showing an increased intensity of CD bands of composites:

To test if we can increase the intensity of CD bands of composites, we prepared D-AuNR@HT5-based composite using M3 as a matrix. The CD and UV-Vis-NIR spectra of this composite are shown in Figure S15. Shortly, this composite showed a stronger CD response in comparison to D-AuHN@HT1-based composite, reflecting the stronger chiroptical response of chiral nanorods, in comparison to chiral nanohelicoids. In regard to the dissymmetry factor, this composite exhibited a decrease of g-factor from 9*10^-3 to 3*10^-3. This experiment proves that using intrinsically chiral NPs with stronger dissymmetry can lead to composites with a stronger CD response.

Supplementary Note 4. NMR analysis of new intermediates and **HT1-HT5** structures.

(9) 4-hydroxyphenyl-4-(hexadecyloxy)benzoate

^{1}H NMR (300 MHz, $CDCl_3$, 25°C, TMS): δ = 8.14 (d, J= 9.0 Hz, 2H; ArH), 7.28 (t, J= 6.2 Hz, 1H; ArH), 6.98 (d, J= 9.0 Hz, 2H; ArH), 6.80-6.71 (m, 3H; ArH), 6.20 (br,1H; OH), 4.06 (t, J= 6.8 Hz, 2H; O$\underline{CH_2}$), 1.89-1.80 (m, 2H), 1.55-1.22 (m, 26H), 0.90 (t, J= 7.0 Hz, 3H, CH_3).

(11) 4-((12-((11-brmoundecanoyl)oxy)dodecyl)oxy)phenyl-4-(hexadecyloxy)benzoate

^{1}H NMR (300 MHz, $CDCl_3$, 25°C, TMS): δ = 8.14 (d, J= 9.0 Hz, 2H; ArH), 7.29 (t, J= 9.0 Hz, 1H; ArH), 6.98 (d, J= 9.0 Hz, 2H; ArH), 6.81-6.74 (m, 3H; ArH), 4.05 (m, 4H; $\underline{CH_2}$OCO and $\underline{CH_2}$O), 3.97 (t, J=7.0 Hz, 2H; O$\underline{CH_2}$), 3.42 (t, J= 7.0 Hz, 2H; $\underline{CH_2}$Br), 2.31 (t, J= 7.8 Hz, 2H; OCO$\underline{CH_2}$), 1.85-1.74 (m, 4H), 1.64-1.58 (m, 6H), 1.40-1.22 (m, 54H), 0.90 (t, J= 7.0 Hz, 3H; CH_3).

(13) 4-[(2,2,2-trichloroethoxy)carbonyl]phenyl-4-(octadec-9-en-1-yloxy)benzoate

^{1}H NMR (300 MHz, CDCl3, 25 °C, TMS): δ = 8.24 (d, J= 9.0 Hz, 2H; ArH), 8.14 (d, J= 9.0 Hz, 2H; ArH), 7.38 (d, J= 9.0 Hz, 2H; ArH), 6.98 (d, J= 9.0 Hz, 2H; ArH), 5.40-5.30 (m, 2H; CH=CH), 4.99 (s, 2H; CH_2CCl_3), 4.05 (t, J = 6.4 Hz, 2H; CH_2O), 1.90-1.76 (m, 2H), 1.51-1.17 (m, 26H), 0.88 (t, J = 6.5 Hz, 3H; CH_3).

(17) 4-[(3-hydroxyphenoxy)carbonyl]phenyl-4-(octadec-9-en-1-yloxy)benzoate

^{1}H NMR (300 MHz, CDCl3, 25 °C, TMS): δ = 8.25 (d, J= 9.0 Hz, 2H; ArH), 8.14 (d, J= 9.0 Hz, 2H; ArH), 7.38 (d, J= 9.0 Hz, 2H; ArH), 7.30-7.26 (m, 1H; ArH), 7.00 (d, J= 9.0 Hz, 2H; ArH), 6.86-6.78 (m, 3H; ArH), 5.40-5.30 (m, 2H; CH=CH), 5.18 (br, 1H; OH), 4.10 (t, J = 6.4 Hz, 2H; $\underline{CH_2}$O), 1.88-1.76 (m, 2H), 1.60-1.20 (m, 26H), 0.90 (t, J = 6.5 Hz, 3H; CH_3).

(HT1) 4-((12-((11-sulfanyloundecanoyl)oxy)dodecyl)oxy)phenyl-4-(octadec-9-en-1-yloxy)benzoate

^{1}H NMR (500 MHz, $CDCl_3$, 25°C, TMS): δ = 8.12 (d, J= 9.0 Hz, 2H; ArH), 7.09 (d, J= 9.0 Hz, 2H; ArH), 6.96 (d, J= 9.0 Hz, 2H; ArH), 6.91 (d, J= 9.0 Hz, 2H; ArH), 5.40-5.32 (m, 2H, $\underline{CH}$=$\underline{CH}$), 4.08-4.01 (m, 4H; $\underline{CH_2}$OCO and $\underline{CH_2}$O), 3.95 (t, J= 7.0 Hz, 2H; $\underline{CH_2}$O), 2.52 (q, J= 7.5 Hz, 2H,

CH2SH), 2.29 (t, J= 7.8 Hz, 2H; CH2CO), 2.05-1.95 (m, 4H), 1.87-1.75 (m, 4H), 1.65-1.55 (m, 6H), 1.50-1.41 (m, 4H), 1.40 (m, 46H), 0.88 (t, J= 7.0 Hz, 3H; CH_3).

^{13}C NMR (500 MHz, $CDCl_3$, 25°C, TMS): δ = 173.98, 165.32, 163.42, 156.75, 144.37, 132.19, 130.00, 129.78, 122.46, 121.68, 115.06, 114.23, 68.39, 68.31, 64.40, 34.40, 34.05, 31.92, 29.78, 29.75, 29.71, 29.67, 29.54, 29.49, 29.46, 29.46, 29.38, 29.37, 29.33, 29.29, 29.25, 29.22, 29.15, 29.11, 29.05, 28.66, 28.37, 27.23, 27.19, 26.04, 25.99, 25.94, 25.02, 24.66, 22.70, 14.13.

(HT2) 4-({4-[(16-sulfanylhexadecanoyl)oxy]phenoxy}carbonyl)phenyl-4-(hexadecyloxy)benzoate

^{1}H NMR (500 MHz, CDCl3, 25 °C, TMS): δ = 8.27 - 8.25 (m, 2H; ArH), 8.16 - 8.14 (m, 2H; ArH), 7.43 (t, J = 8.0 Hz, 1H; ArH), 7.38 - 7.35 (m, 2 H; ArH), 7.13 - 7.11 (m, 1H; ArH), 7.05 - 7.02 (m, 2H; ArH), 7.00-6.98 (m, 2H; ArH), 4.05 (t, J = 7.0 Hz, 2H), 2.56 (t, J = 7.5 Hz, 2H; CH2COO), 2.52 (q, J = 7.0 Hz, 2H; CH2S), 1.88 - 1.81 (m, 2H; CH2CH2O), 1.49 - 1.26 (m, 53H), 0.88 (t, J = 7.0 Hz, 3H; CH3).

^{13}C NMR (500 MHz, CDCl3, 25 °C, TMS): δ = 171.98, 164.31, 164.08, 163.84, 155.47, 151.31, 132.43, 131.83, 129.75, 129.76, 126.62, 122.15, 120.91, 119.15, 119.04, 115.66, 114.42, 45.21, 34.38, 34.08, 32.66, 31.93, 29.70, 29.68, 29.67, 29.63, 29.61, 29.56, 29.47, 29.40, 29.37, 29.34, 29.10, 28.90, 28.77, 28.19, 26.90, 25.98, 24.89, 22.70, 14.14

(HT3) 4-((12-((11-sulfanyloundecanoyl)oxy)dodecyl)oxy)phenyl-4-(hexadecyloxy)benzoate

^{1}H NMR (500 MHz, $CDCl_3$, 25°C, TMS): δ = 8.12 (d, J= 9.0 Hz, 2H; ArH), 7.29 (t, J= 7.4 Hz, 1H; ArH), 6.96 (d, J= 9.0 Hz, 2H; ArH), 6.81-6.74 (m, 3H; ArH), 4.08-4.02 (m, 4H; CH2OCO and CH2O), 3.95 (t, J=7.2 Hz, 2H; OCH2), 2.52 (q, J= 7.2 Hz, 2H; CH2SH), 2.29 (t, J= 7.0 Hz, 2H;

OCO$\underline{CH_2}$), 1.85-1.74 (m, 4H), 1.64-1.58 (m, 6H), 1.51-1.40 (m, 4H), 1.40-1.22 (m, 50H), 0.88 (t, J= 7.0 Hz, 3H; CH_3).

^{13}C NMR (500 MHz, $CDCl_3$, 25°C, TMS): δ = 174.01, 164.90, 163.53, 160.10, 152.04, 132.27, 129.74, 121.59, 114.29, 113.79, 112.23, 108.82, 68.35, 68.17, 64.42, 34.41, 34.06, 31.95, 19.72, 29.70, 29.68, 29.61, 29.58, 29.49, 29.47, 29.46, 29.39, 29.36, 29.25, 29.21, 29.15, 29.12, 29.06, 28.67, 28.38, 25.03, 24.67, 22.72, 14.14.

(HT4) 4-({4-[(16-sulfanylhexadecanoyl)oxy]phenoxy}carbonyl)phenyl-4- (octadec-9-en-1-yloxy))benzoate

^{1}H NMR (500 MHz, $CDCl_3$, 25°C, TMS): δ = 8.26 (d, J= 9.0 Hz, 2H; ArH), 8.15 (d, J= 9.0 Hz, 2H; ArH), 7.43 (t, J= 8.5 Hz, 1H; ArH), 7.37 (d, J= 9.0 Hz, 2H; ArH), 7.14-7.11 (m, 1H; ArH), 7.06-7.01 (m, 2H; ArH), 6.99 (d, J= 9.0 Hz, 2H; ArH), 5.40-5.30 (m, 2H, CH=CH), 4.05 (t, J= 8.4 Hz, 2H; O$\underline{CH_2}$), 2.57-2.49 (m, 4H; $\underline{CH_2}$SH and OCO$\underline{CH_2}$), 2.06-1.95 (m, 4H), 1.86-1.79 (m, 2H), 1.78-1.70 (m, 2H), 1.65-1.20 (m, 46H), 0.88 (t, J= 7.0 Hz, 3H; CH_3).

^{13}C NMR (500 MHz, $CDCl_3$, 25°C, TMS): δ = 171.94, 164.33, 164.10, 163.85, 155.49, 151.33, 132.45, 131.85, 130.04, 129.80, 126.65, 122.16, 120.95, 119.17, 119.05, 115.68, 114.44, 68.40, 34.41, 34.09, 31.94, 29.80, 29.76, 29.67, 29.66, 29.61, 29.55, 29.47, 29.36, 29.28, 29.24, 29.12, 29.11, 28.42, 27.25, 27.21, 26.00, 24.91, 24.69, 22.71, 14.15

(HT5) 4-[(16-sulfanylhexadecanoyl)oxy]phenoxy -4-(octadec-9-en-1-yloxy)benzoate

^{1}H NMR (500 MHz, CDCl3, 25 °C, TMS): δ = 8.15 (d, 2H; J= 8.6 Hz; ArH), 7.21 (d, 2H; J= 8.6 Hz; ArH), 7.16 (d, 2H; J= 8.6 Hz; ArH), 6.96 (d, 2H; J= 8.6 Hz; ArH), 5.39-5.36 (m, 2H,

CH=CH), 4.06 (t, 2H; J= 7.0 Hz; CH_2O), 2.56-2.49 (m, 4H; CH_2SH and $COOCH_2$), 2.18-2.02 (m, 4H), 1.85 - 1.75 (m, 4H), 1.65 - 1.30 (m, 46H), 0.90 (t, 3H; J = 7.0 Hz; CH_3).

^{13}C NMR (500 MHz, $CDCl_3$, 25 °C, TMS): δ = 173.10, 163.32, 162.35, 1630.85, 158.46, 155.37, 151.84, 132.35, 131.80, 129.82, 126.95, 124.38, 122.10, 120.96, 114.43, 113.40, 112.42, 108.16, 68.41, 68.21, 64.41, 34.41, 34.06, 31.95, 29.72, 29.70, 29.68, 29.61, 29.58, 29.50, 29.47, 29.46, 29.39, 29.37, 29.26, 29.21, 29.16, 29.11, 29.06, 28.62, 28.30, 26.02, 26.00, 25.86, 25.03, 24.67, 22.70, 14.15.

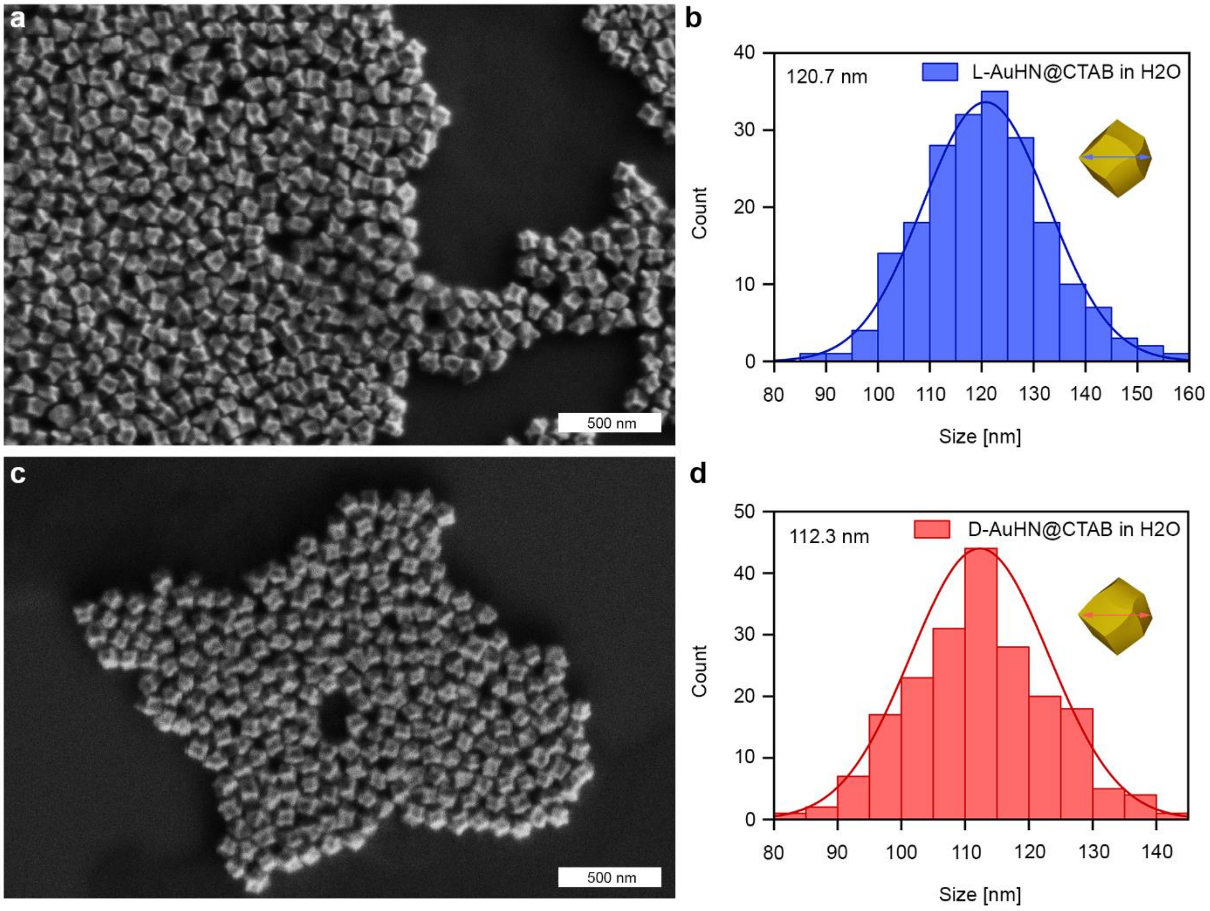

Figure S1. SEM micrographs of (a) L-AuHN@CTAB (c) D-AuHN@CTAB nanoparticles dropcasted from water dispersions and corresponding statistical analysis of nanoparticle diameters (b) L-AuHN@CTAB (mean ~121 nm) (d) D-AuHN@CTAB (mean ~112 nm). Size measurements are reported based on the diameter of a sphere circumscribed on chiral nanoparticles.

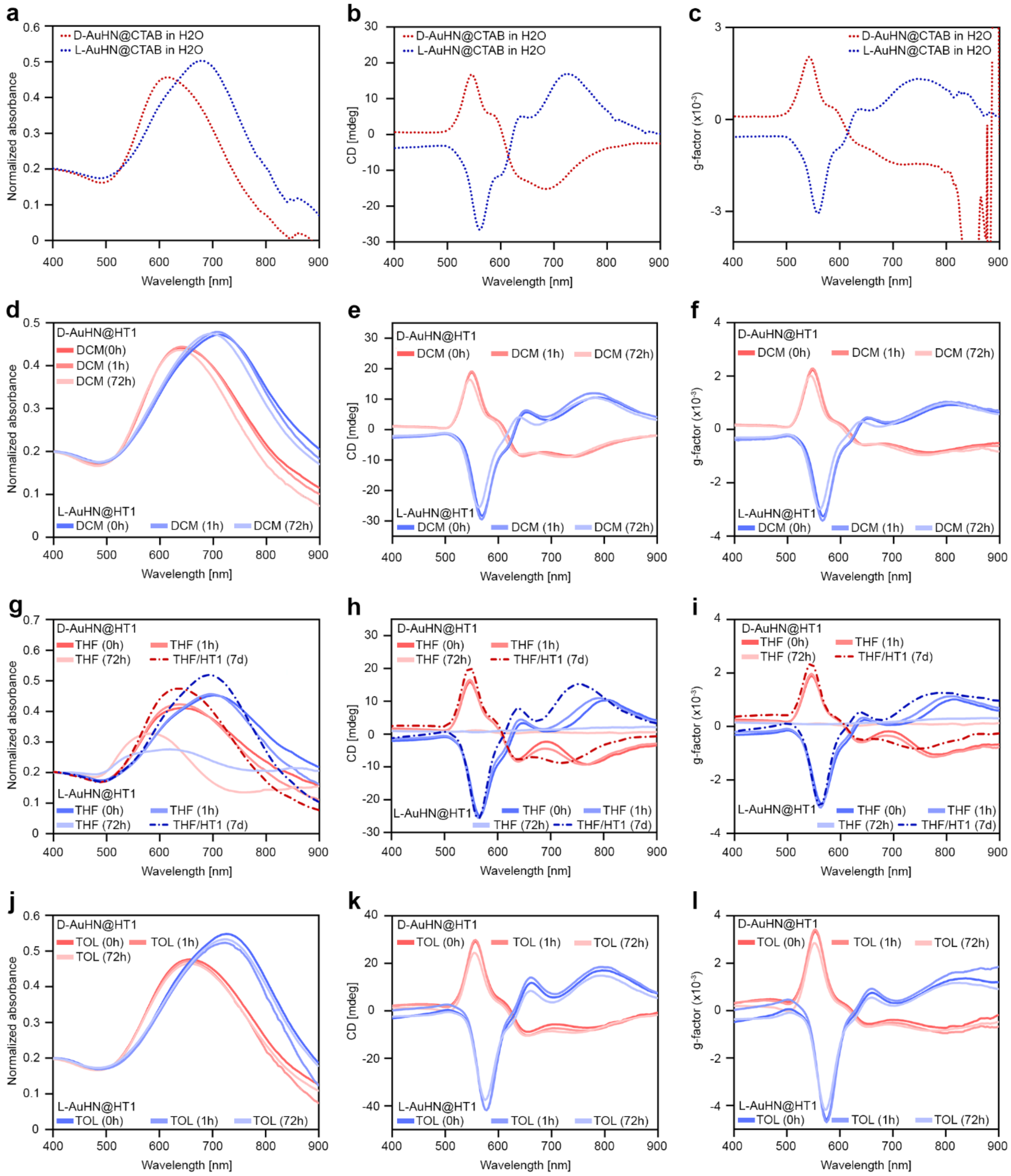

Figure S2. Comparison of chiral, hydrophobic NPs stability in different solvents. Absorption, CD and g-factor spectra of AuHN dispersed in (a-c) water, (d-f) dichloromethane, (g-i) tetrahydrofuran, and (j-l) toluene. All spectra were recorded at a given time interval from the phase transfer process (0h, 1h, 72h). Vis-NIR spectra were normalized to 0.2 absorbance value at 400 nm to remove concentration dependence and emphasize the influence of solvent and time.

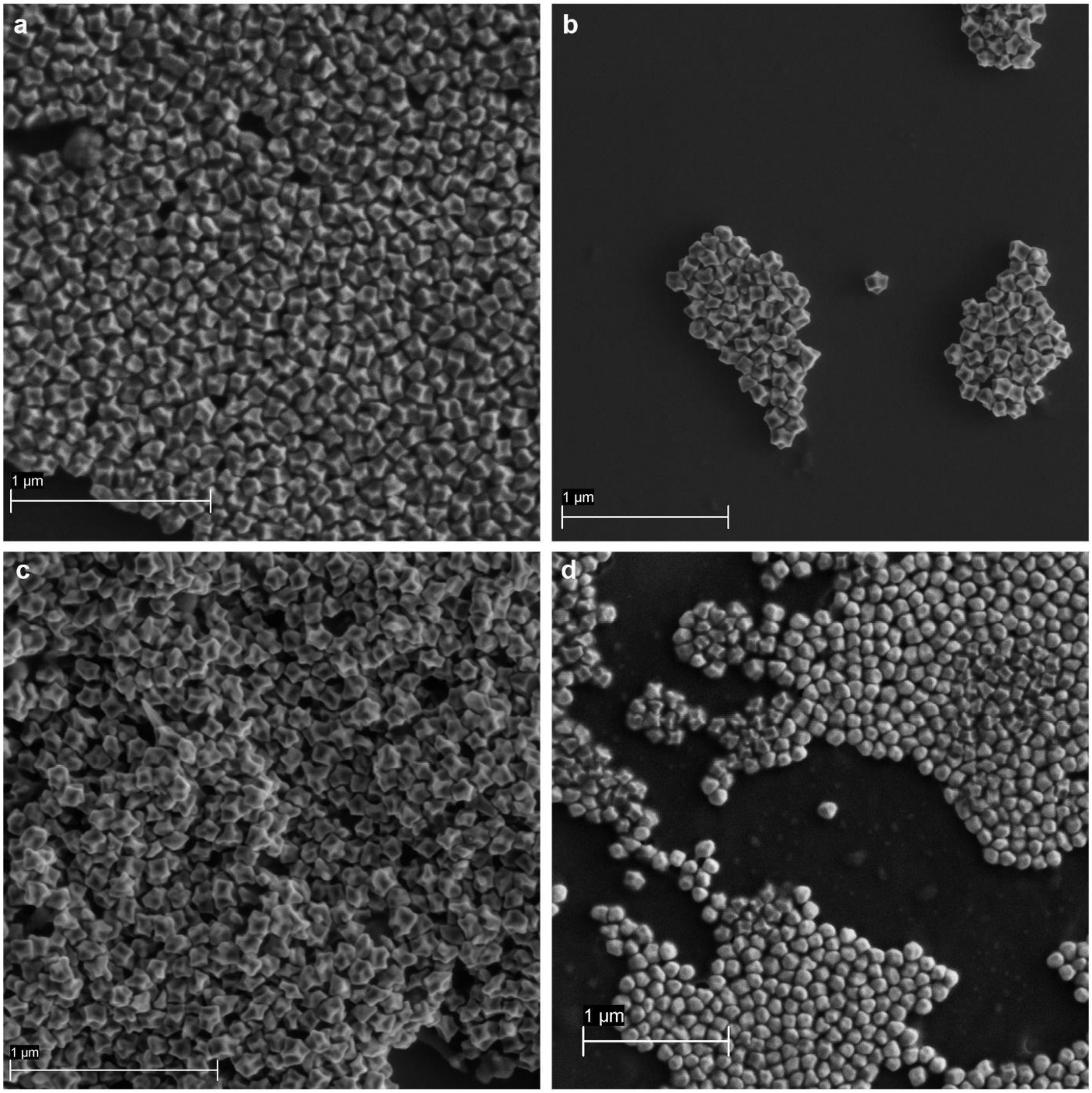

Figure S3. SEM images of L-AuHN@HT1 dropcasted from different solvents. (a) dichloromethane, (b) toluene, (c) tetrahydrofuran (sample prepared directly after the phase transfer), (d) tetrahydrofuran (sample prepared after storing NPs for 3 days, without HT1 excess).

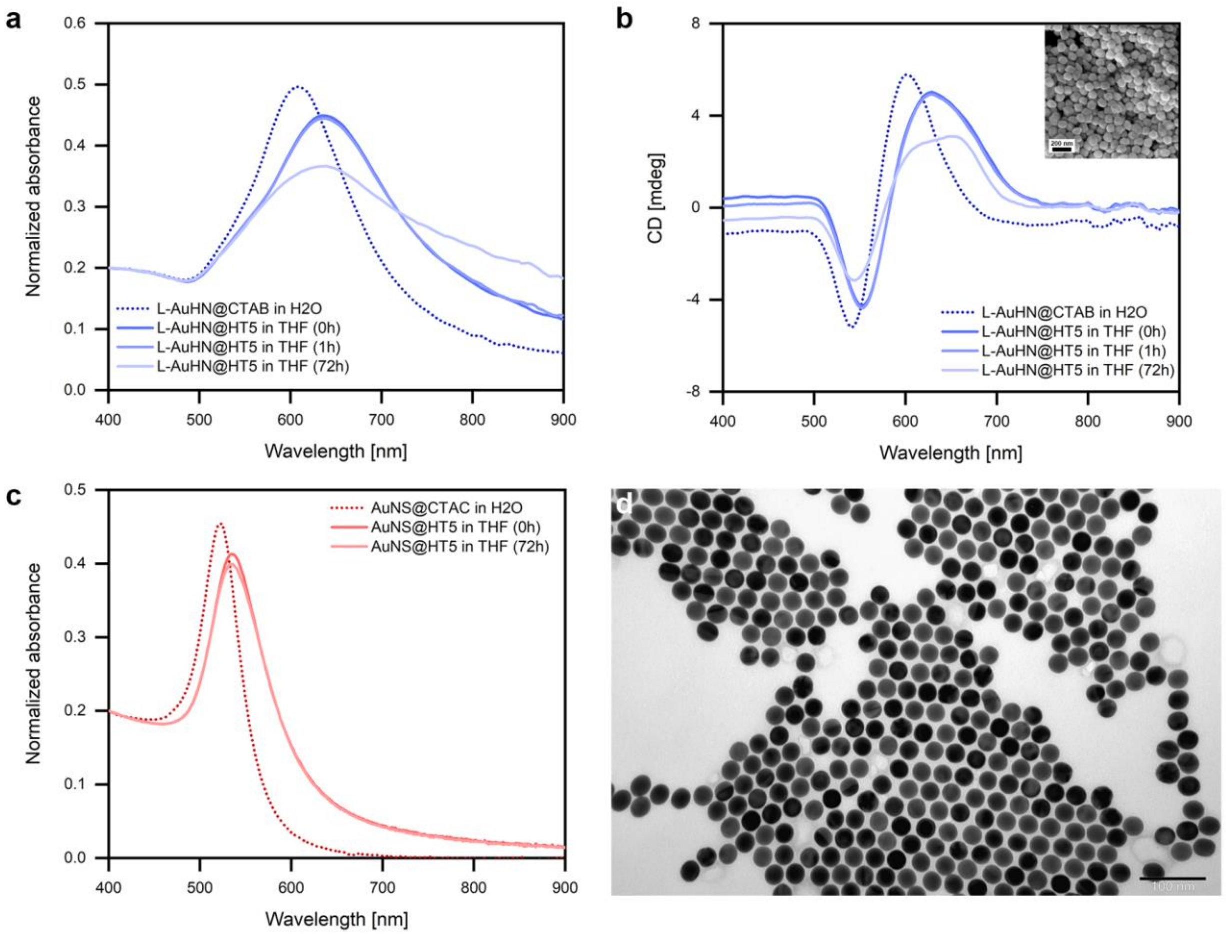

Figure S4. Comparison of stability of chiral and spherical Au NPs stored in THF without excess of a hydrophobic thiol. (a) Vis-NIR and (b) CD spectra of L-AuHN@CTAB dispersed in water and L-AuHN@HT5 dispersed in tetrahydrofuran. SEM image of L-AuHN@HT5 stored in THF for 72 hours is shown in insert. (c) Vis-NIR spectra of gold nanospheres AuNS@CTAC dispersed in water and AuNS@HT5 dispersed in tetrahydrofuran. (d) TEM images of AuNS@CTAC with an average diameter of 31±2 nm.

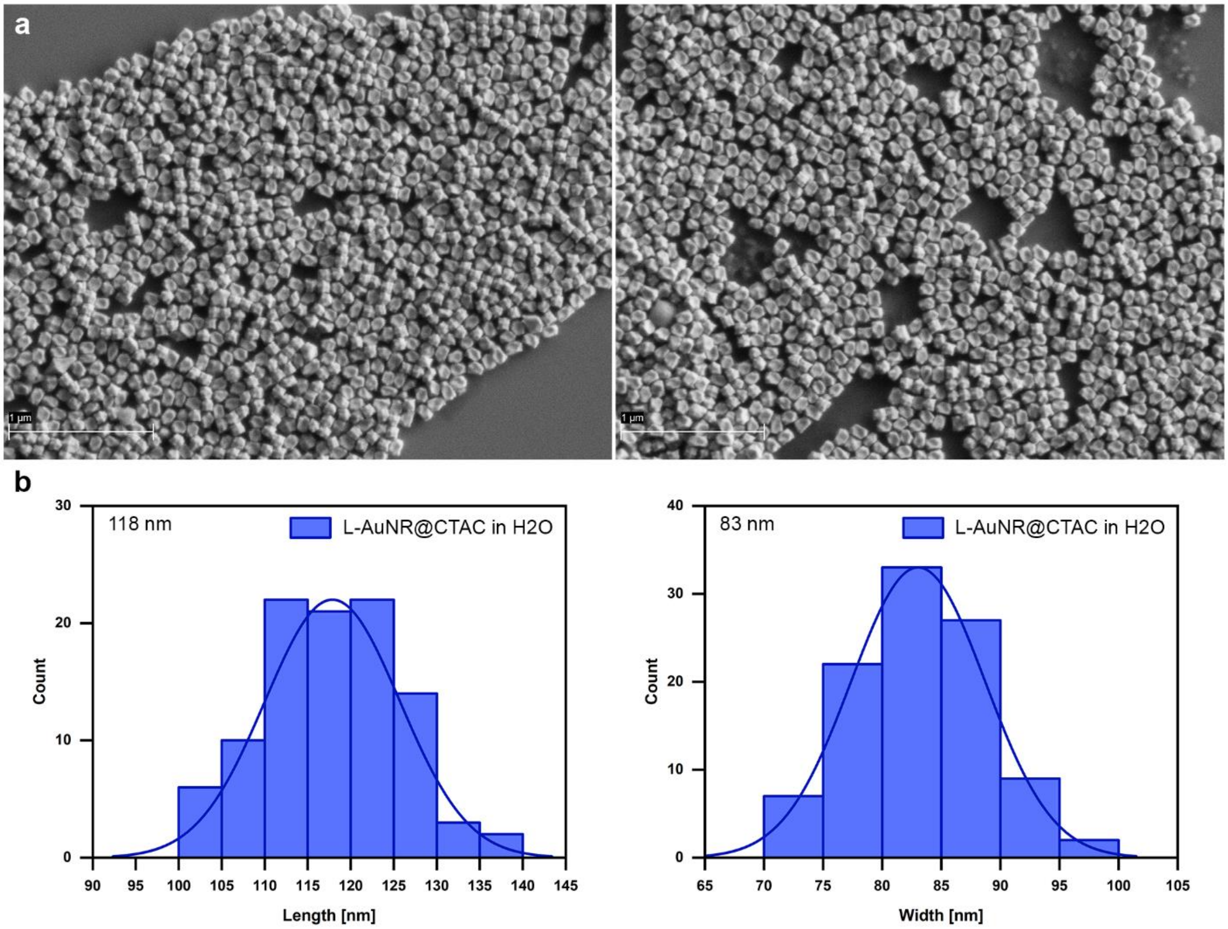

Figure S5. (a) SEM micrographs and (b) corresponding statistical analysis of the length (left) and width (right) of the L-AuNR@CTAC nanoparticles (dropcasted from water).

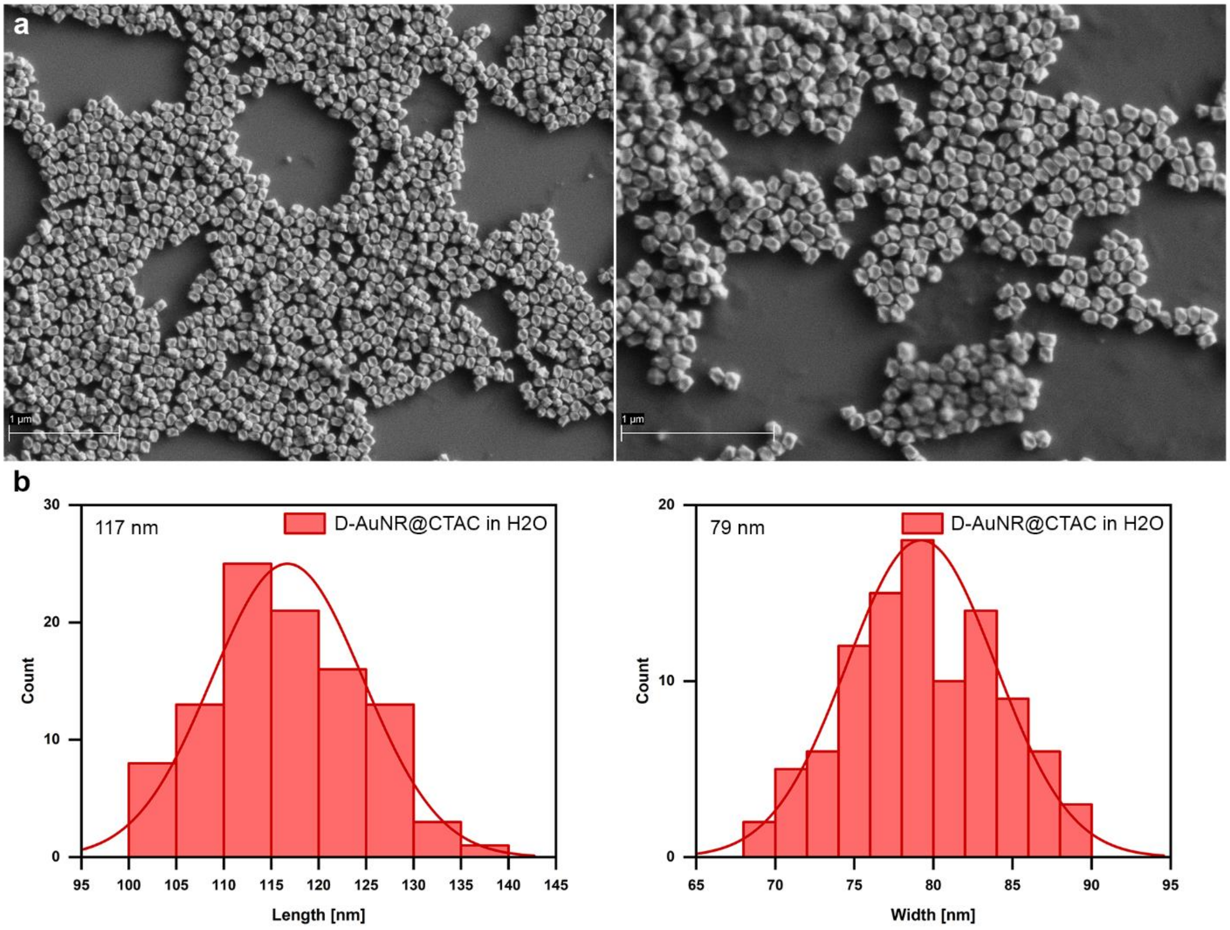

Figure S6. (a) SEM micrographs and corresponding (b) statistical analysis of the length (left) and width (right) of the D-AuNR@CTAC nanoparticles (dropcasted from water).

Table S1. The Vis-NIR absorption analysis. Transversal LSPR band position of L-AuNR and D-AuNR NPs stabilized with CTAC and different hydrophobic thiols; samples were dispersed in water and DCM, respectively.

	**L-AuNR**	**D-AuNR**
	**Transversal LSPR**	
**CTAC**	605 nm	595 nm
**HT2**	623 nm	615 nm
**HT3**	622 nm	610 nm
**HT4**	624 nm	614 nm
**HT5**	626 nm	618 nm

Table S2. The circular dichroism analysis. The first and second Cotton band position for L-AuNR and D-AuNRs stabilized with CTAC and different hydrophobic thiols, dispersed in water and DCM, respectively.

	**L-AuNR**		**D-AuNR**	
	**First Cotton band position**	**Second Cotton band position**	**First Cotton band position**	**Second Cotton band position**
**CTAC**	585 nm	685 nm	580 nm	680 nm
**HT2**	600 nm	724 nm	595 nm	729 nm
**HT3**	597 nm	726 nm	594 nm	727 nm
**HT4**	599 nm	709 nm	596 nm	705 nm
**HT5**	604 nm	713 nm	600 nm	706 nm

Table S3. The dissymmetry coefficient value (g-factor) analysis. The g-factor values for L-AuNR and D-AuNRs stabilized with CTAC and different hydrophobic thiols, dispersed in water and DCM, respectively, calculated for the first cotton band.

	**G-factor (calculated for the first Cotton band)**	
	**L-AuNR**	**D-AuNR**
**CTAC**	-0.00862	0.00939
**HT2**	-0.00955	0.00881
**HT3**	-0.0087	0.00898
**HT4**	-0.00853	0.00999
**HT5**	-0.00951	0.00837

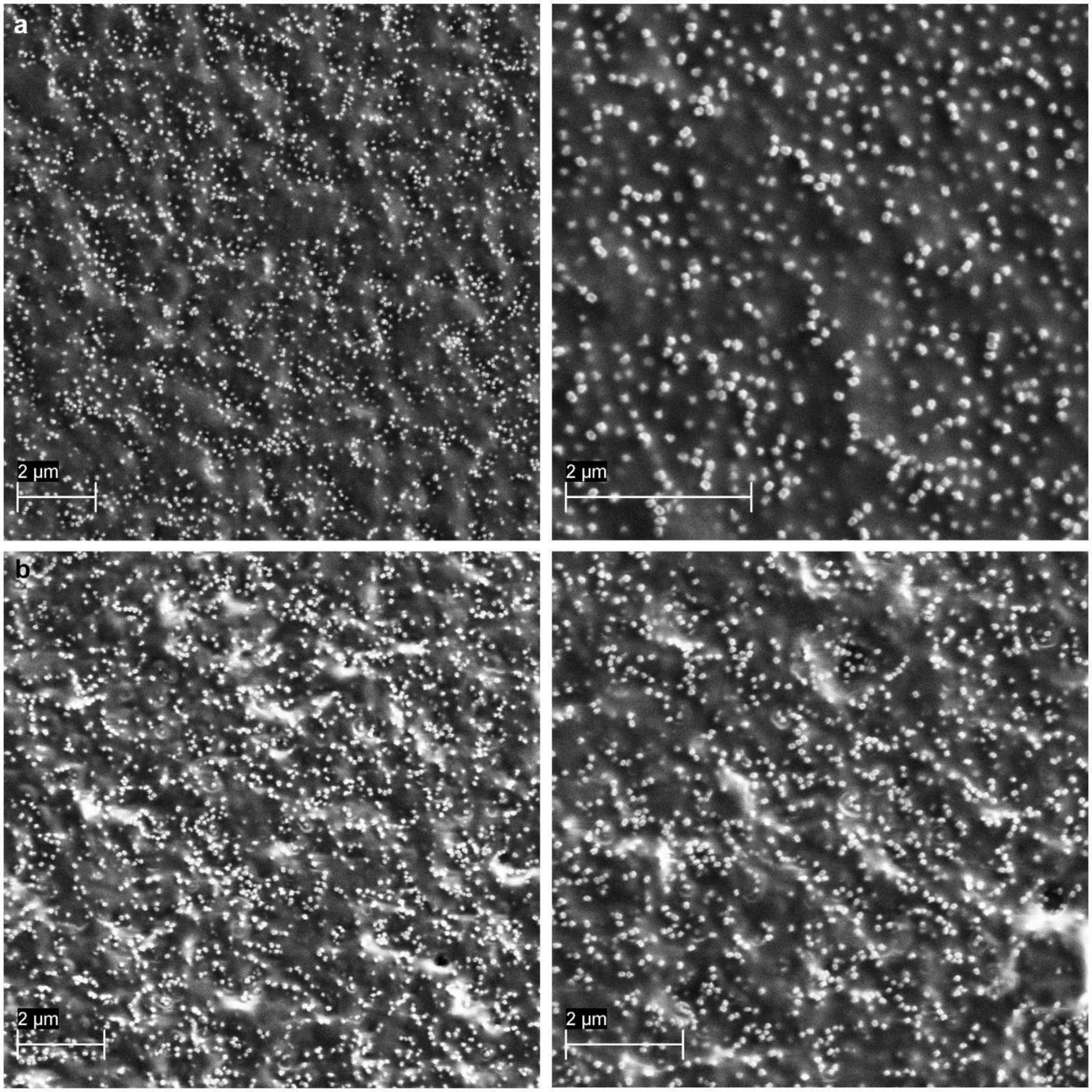

Figure S7. SEM micrographs of (a) D-AuNR@HT2 and (b) L-AuNR@HT2 stored in the excess of free hydrophobic thiol; dropcasted from DCM.

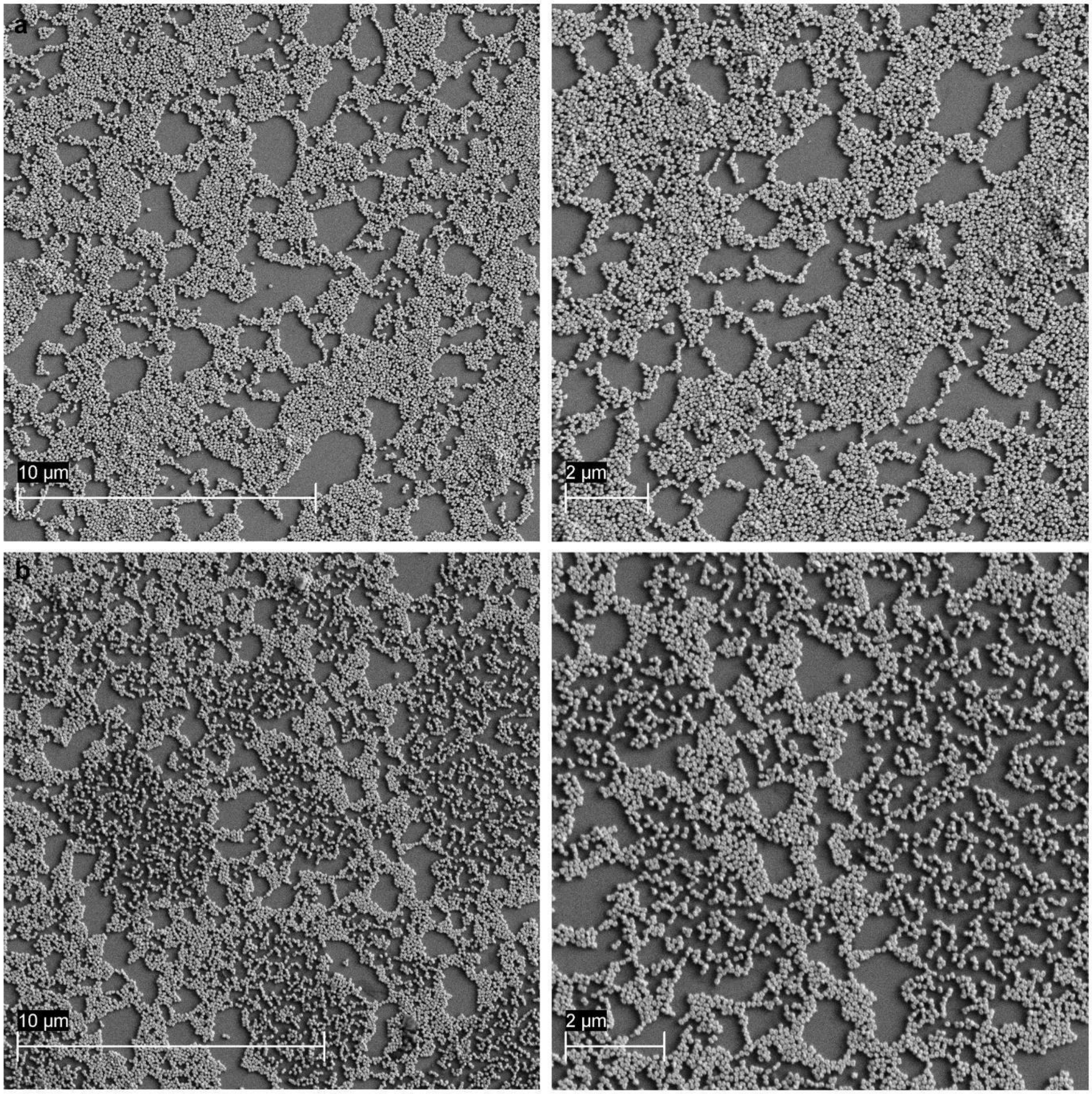

Figure S8. SEM micrographs of (a) D-AuNR@HT2 and (b) L-AuNR@HT2 fully purified from the excess of hydrophobic thiol; dropcasted from DCM.

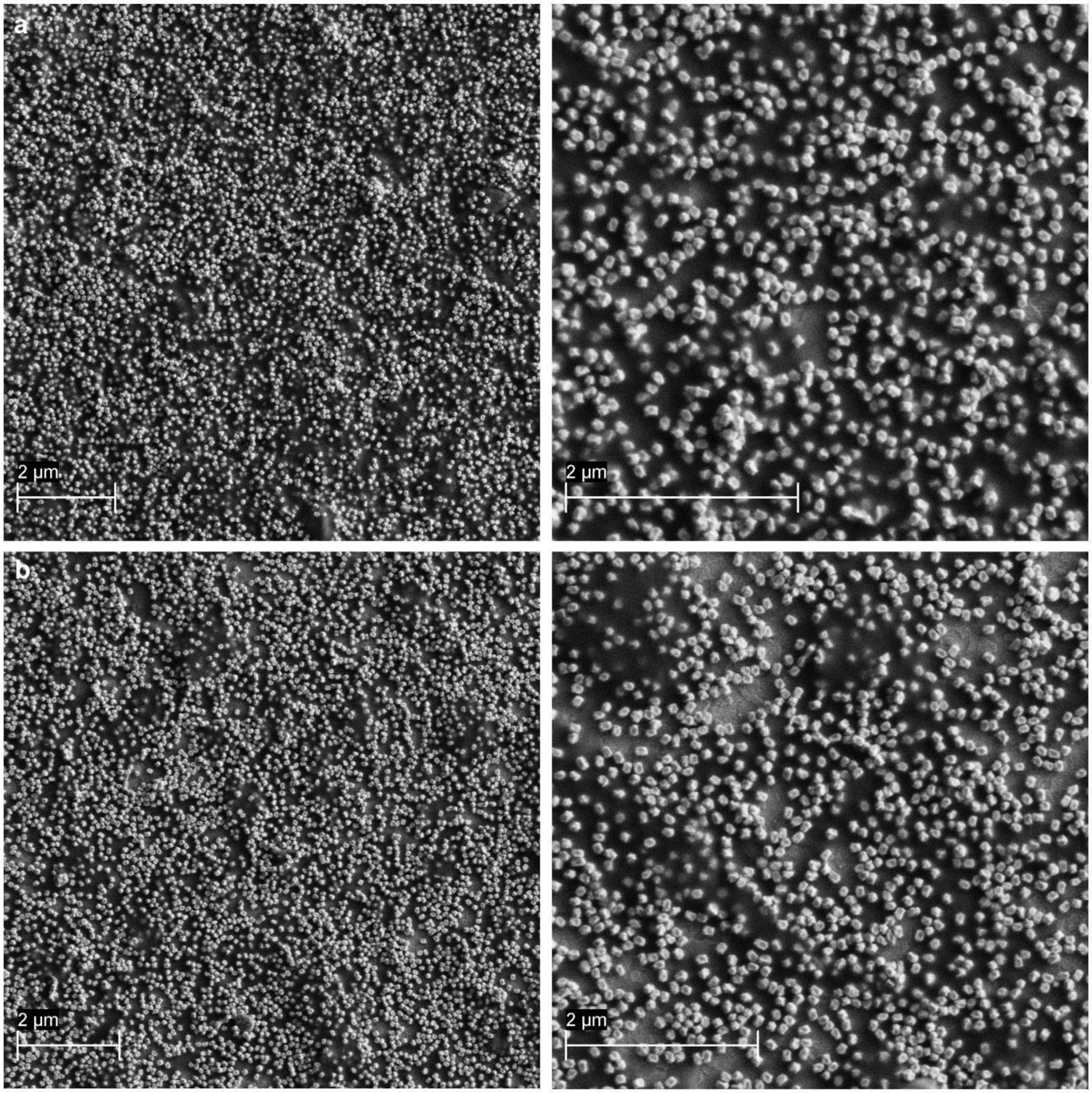

Figure S9. SEM micrographs of (a) D-AuNR@HT2 and (b) L-AuNR@HT2 stored in the excess of hydrophobic thiol; dropcasted from THF.

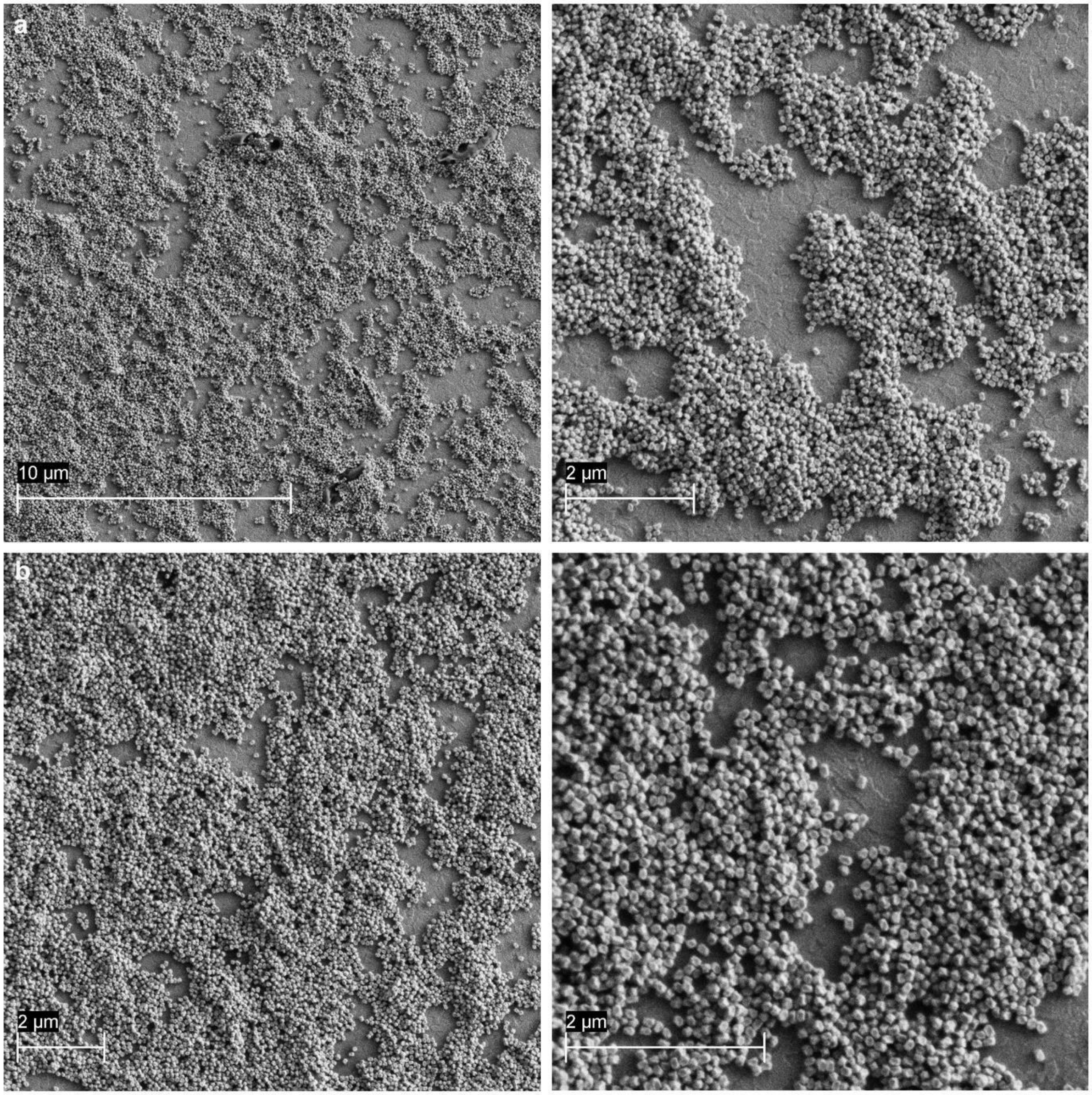

Figure S10. SEM micrographs of (a) D-AuNR@HT2 and (b) L-AuNR@HT2 fully purified from the excess of hydrophobic thiol; dropcasted from THF.

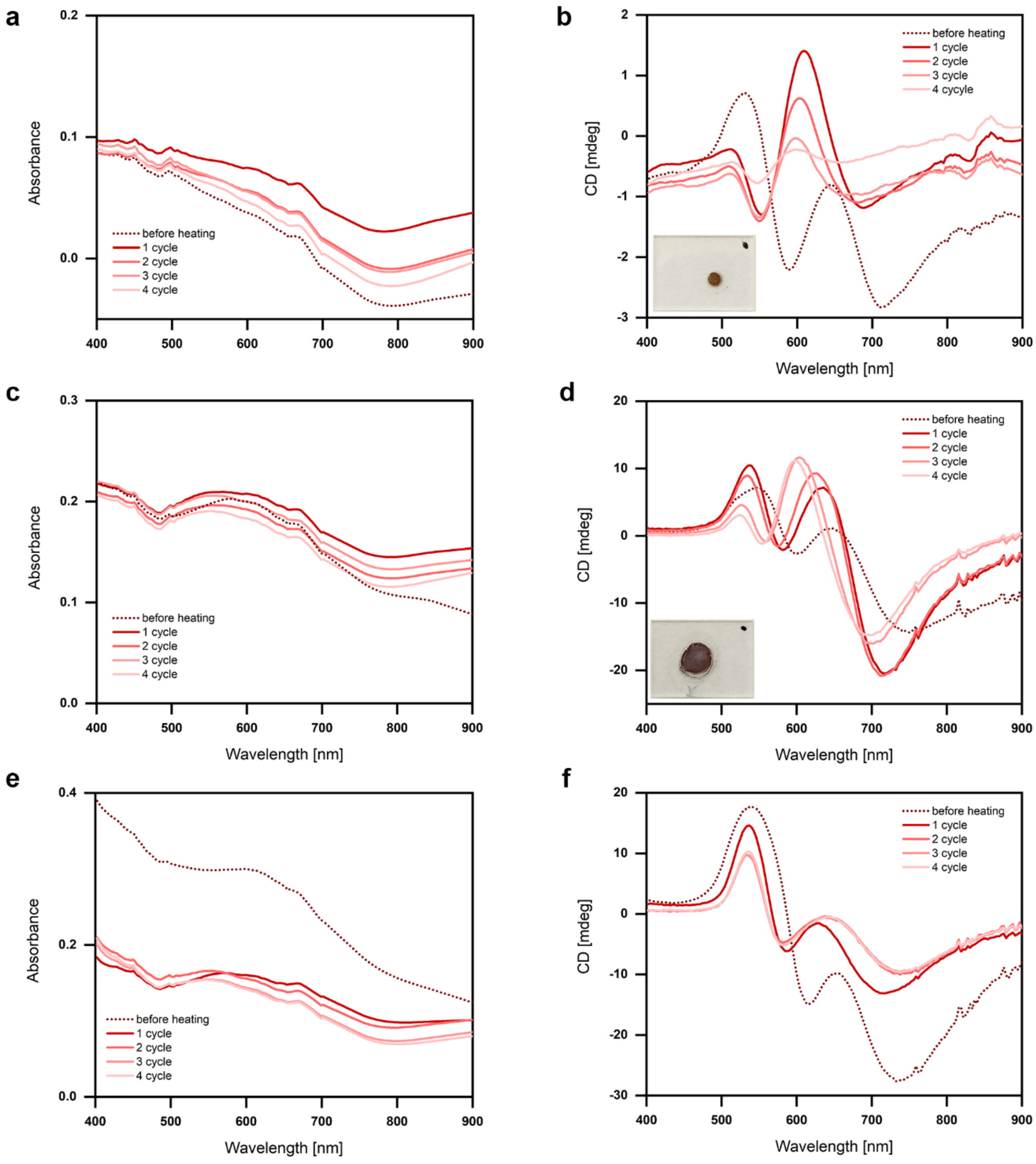

Figure S11. Thermal stability test of D-AuNR@HT2. Absorption and CD spectra of (a-b) purified nanoparticles, (c-d) nanoparticles with HT2 excess, (e-f) nanoparticles mixed with M1 matrix (D-AuNR@HT2 in M1) dropcasted onto ITO coated glass substrate from DCM. For each sample, 4 heating-cooling cycles were performed.

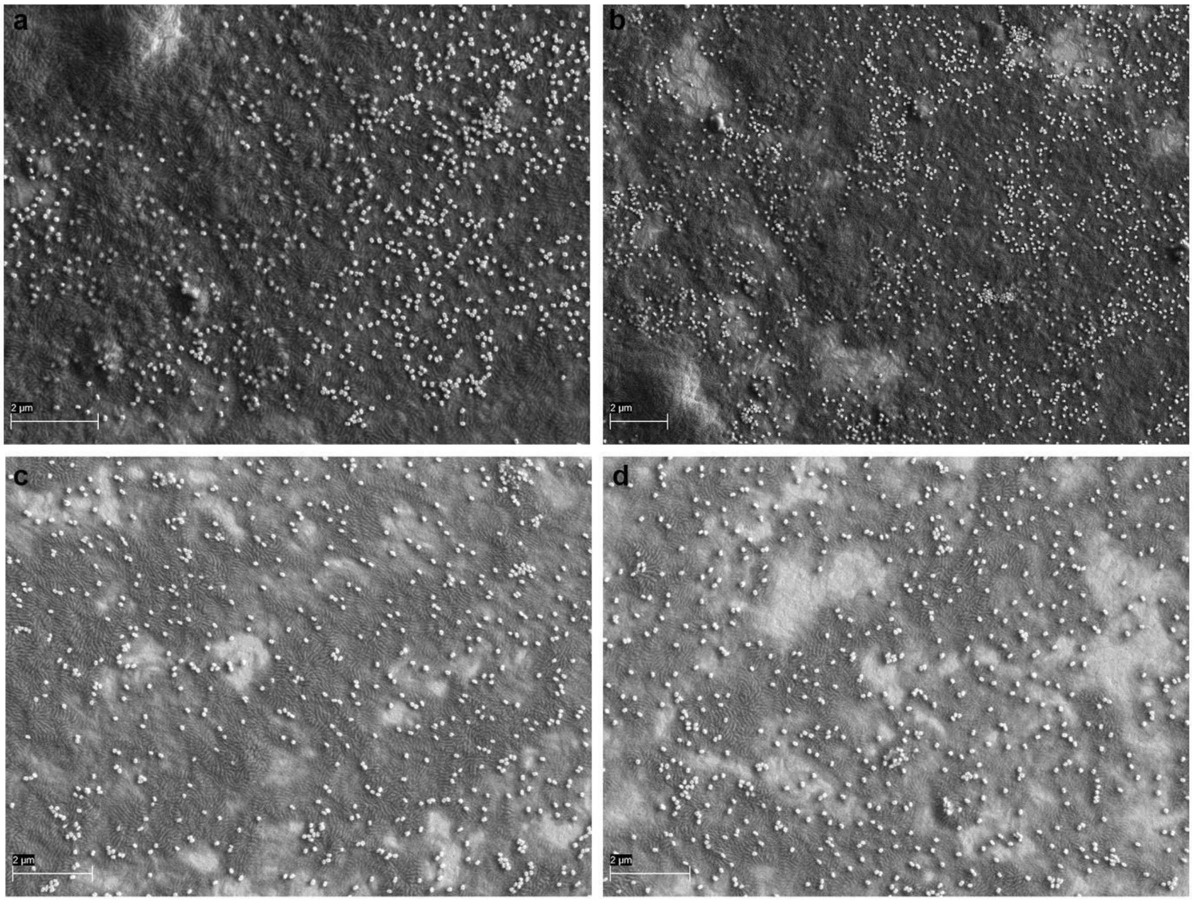

Figure S12. SEM micrographs of D-AuNR@HT2 in M1 (a-b) 1:1 AuNR@HT2 to M1 mass ratio (c-d) 1:2 AuNR@HT2 to M2 mass ratio. SEM analysis showed ~ 23% nanorods assemble in a side-by-side manner and ~7% assembled tip-to-tip. This fact indicate that LSPR and PCD shift and broadening may be caused by partial plasmon coupling.[2]

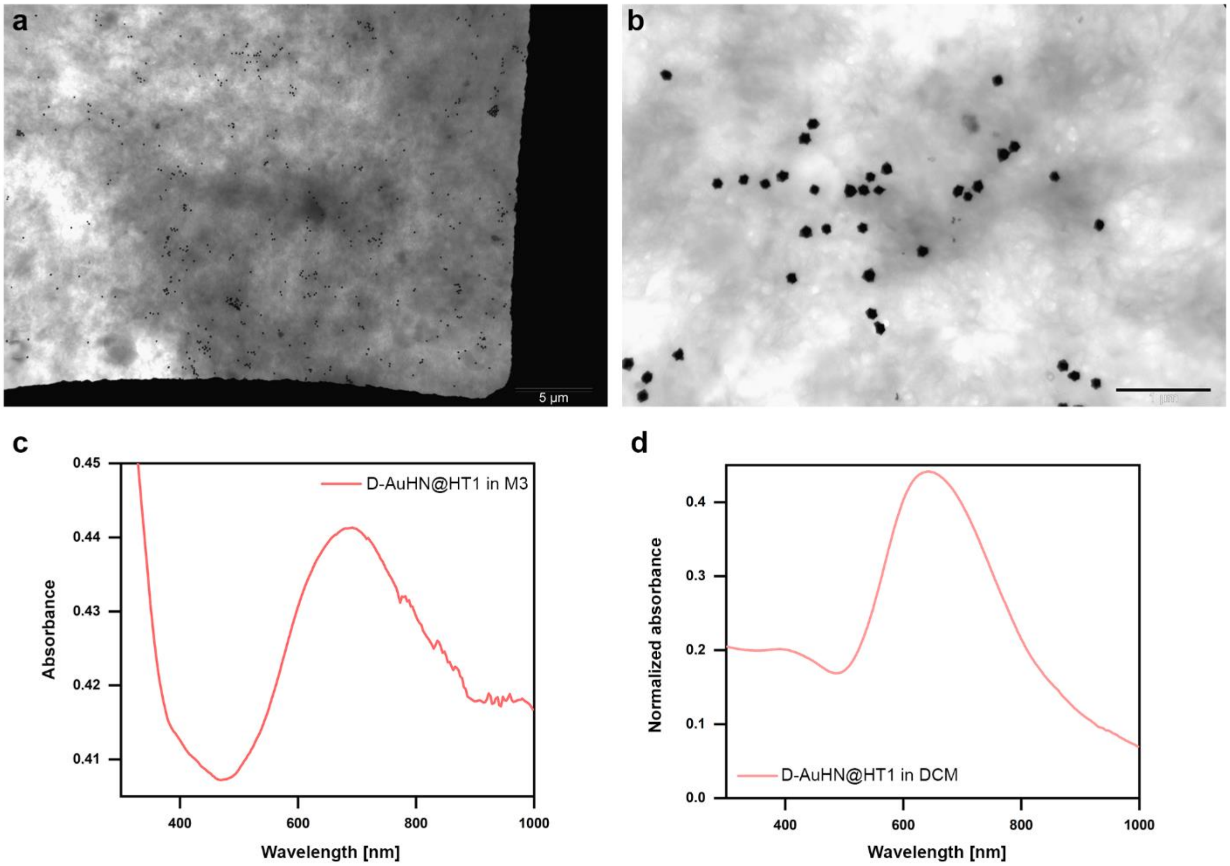

Figure S13. (a-b) TEM micrographs of D-AuHN@HT1 in M2. (c) The UV-Vis-NIR spectrum of D-AuHN@HT1 in M2 nanocomposite. (d) The UV-Vis-NIR spectrum of D-AuHN@HT1 in DCM.

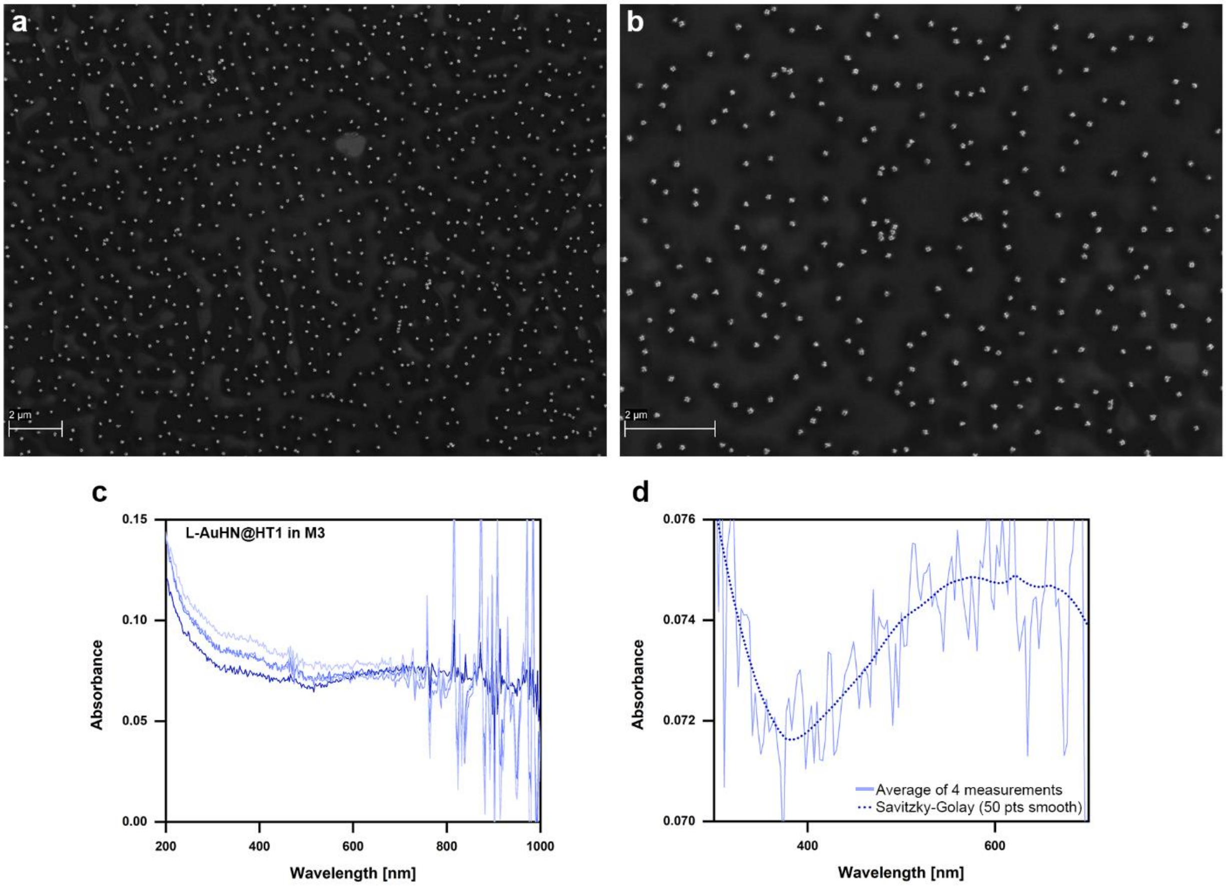

Figure S14. (a-b) SEM micrographs of L-AuHN@HT1 in M3. (c) UV-Vis-NIR spectra of L-AuHN@HT1 in M3 nanocomposite, measured in four different parts of the sample. (d) A solid line for eye guidance is an average of measurements shown in c (Savitzky-Golay, 50 pts smooth).

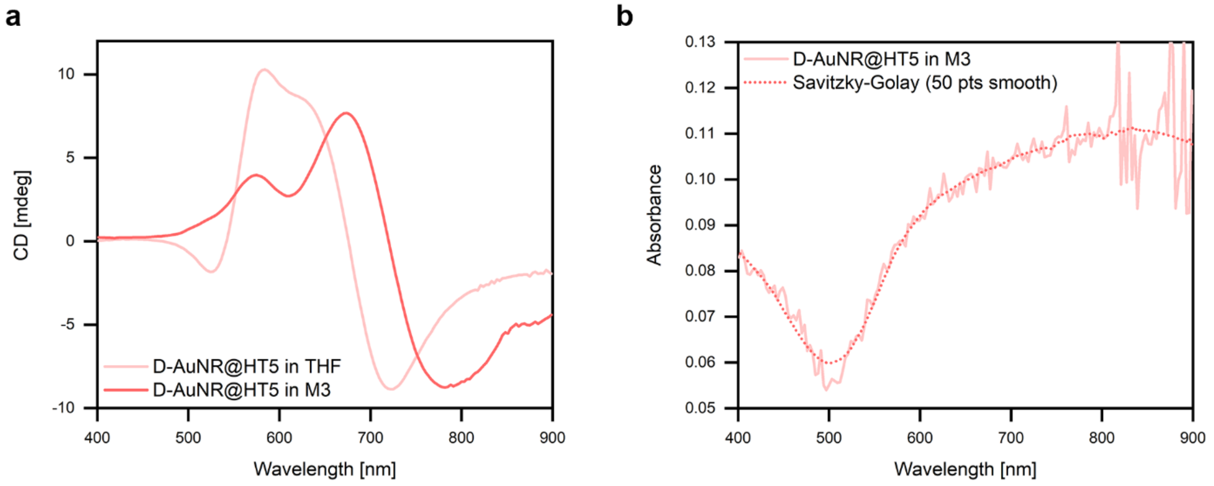

Figure S15. (a) CD spectra of D-AuNR@HT5 in M3 composite dropcasted onto a solid substrate compared to CD spectra of D-AuNR@HT5 dispersion in THF (c) Vis-NIR spectra of D-AuNR@HT5 in M3 nanocomposite. A dotted line is for eye guidance (Savitzky-Golay, 50 pts smooth).

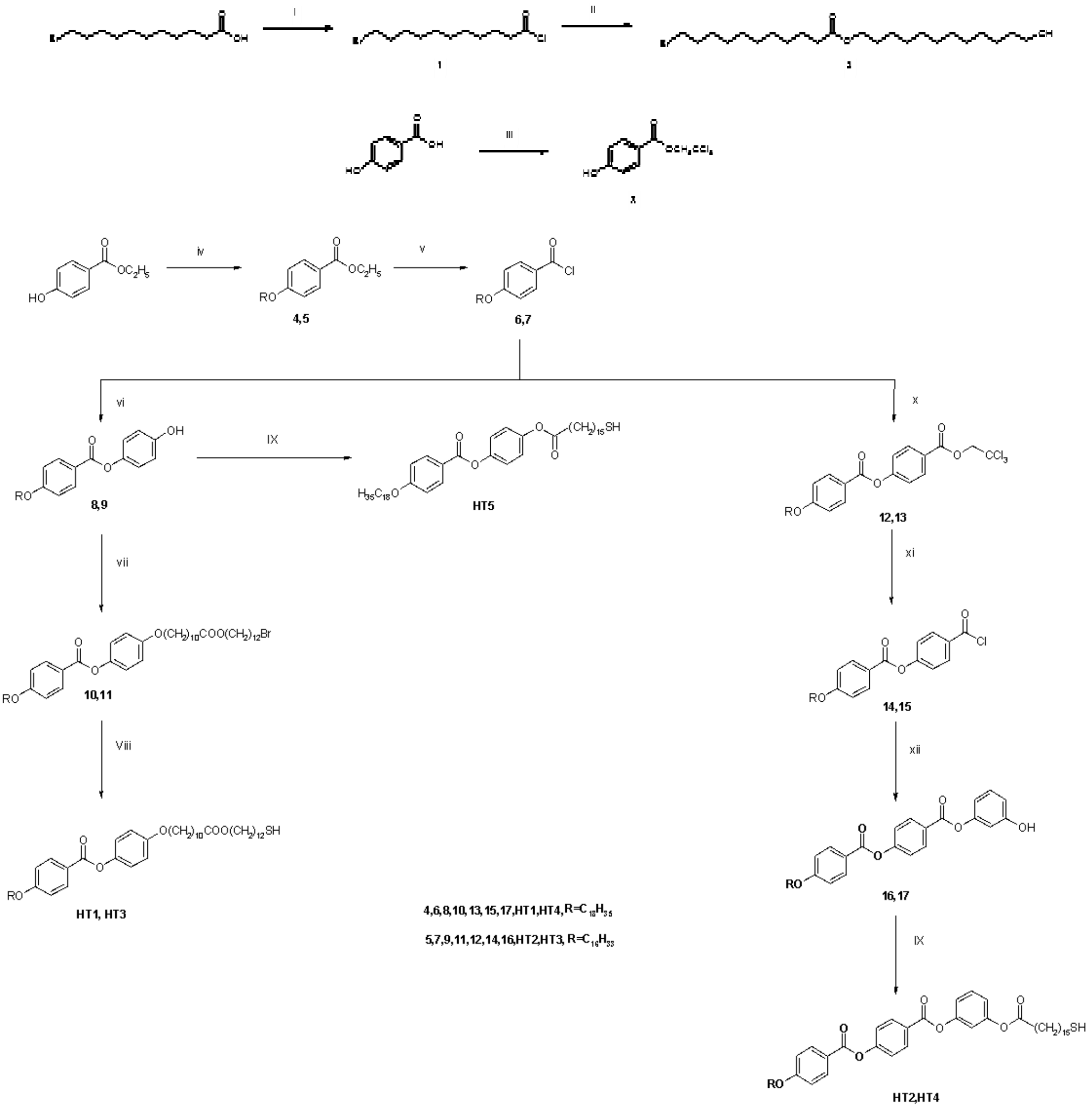

Figure S16. Synthetic route for the preparation of (pro)mesogenic ligands (HT1-HT5).

Reagents and conditions: (i) oxalyl chloride, DMF, DCM; (ii) 1,12-dodecanediol, pyridine, DMAP, THF; (iii) 2,2,2-trichloroethanol, $NaHCO_3$; (iv) oleyl alcohol or cetyl alcohol, PPh_3, DIAD, THF, ultrasounds; (v) a. KOH, ethanol, rfx.; b. oxalyl chloride, toluene, rfx.; (vi) hydroquinone, TEA, DMAP, THF, rfx.; (vii) compound **2**, PPh_3, DIAD, THF, ultrasounds; (viii) HMDST, TBAF, THF; (ix) 16-mercaptohexadecanoic acid, DCC, DMAP, DCM; (x) compound

3, TEA, DMAP, THF, rfx. ; (xi) a.Zn, CH_3COOH, THF, rfx. b. oxalyl chloride, toluene, rfx.; (xii) resorcinol, TEA, DMAP, THF, rfx.

Ligands **HT1**, **HT2** and **HT5** (as well as all intermediate compounds) were obtained according to procedures described in our previous works.[3-5]

Compound **HT3** was obtained according to analogous procedure to synthesis of the compound **HT1** using cetyl alcohol instead oleyl alcohol in step iv.

Ligand **HT4** was obtained according to analogous procedure to synthesis of the compound **HT2** using oleyl alcohol instead cetyl alcohol in step iv.

References

(1) Lu, J.; Xue,Y.; Bernardino, K.; Zhang N. N.; Gomes, W.R.; Ramesar, N.S.; Liu, S.; Hu, Z.; Sun, T.; de Moura, A.f.; Kotov, N.A.; Liu, K. Enhanced optical asymmetry in supramolecular chiroplasmonic assemblies with long-range order. *Science* **2021**, 371, 6536, 1368-1374. 10.1126/science.abd8576

(2) Zheng, G.; Bao, Z.; Pérez-Juste, J.; Du, R.; Liu, W.; Dai, J.; Zhang, W.; Lee, L. Y. S.; Wong, K. Y. Tuning the Morphology and Chiroptical Properties of Discrete Gold Nanorods with Amino Acids. *Angew. Chemie - Int. Ed.* **2018**, *57* (50), 16452–16457. 10.1002/anie.201810693.

(3) Bagiński, M.; Tupikowska, M.; González-Rubio, G.; Wójcik, M.; Lewandowski, W. Shaping Liquid Crystals with Gold Nanoparticles: Helical Assemblies with Tunable and Hierarchical Structures Via Thin-Film Cooperative Interactions. *Adv. Mater.* **2020**, *32*, No. 1904581. 10.1002/adma.201904581.

(4) Lewandowski, W.; Fruhnert, M.; Mieczkowski, J.; Rockstuhl C.; Gorecka E. Dynamically self-assembled silver nanoparticles as a thermally tunable metamaterial. *Nat Commun* **2015,** 6**,** 6590. 10.1038/ncomms7590

(5) Grzelak, D.; Tupikowska, M.; Vila-Liarte, D.; Beutel, D.; Bagiński, M.; Parzyszek, S.; Góra, M.; Rockstuhl, C.; Liz-Marzán, L. M.; Lewandowski, W. Liquid Crystal Templated Chiral Plasmonic Films with Dynamic Tunability and Moldability. *Adv. Funct. Mater.* **2022**, 32, No. 2111280. 10.1002/adfm.202111280
